# Much of a Muchness: On the Origins of Two- and Three-Photon Absorption Activity of Dipolar Y-Shaped Chromophores

**DOI:** 10.1021/acs.jpca.1c10098

**Published:** 2022-01-27

**Authors:** Marta Chołuj, Rojalini Behera, Elizaveta F. Petrusevich, Wojciech Bartkowiak, Md. Mehboob Alam, Robert Zaleśny

**Affiliations:** † Faculty of Chemistry, Wrocław University of Science and Technology, Wyb. Wyspiańskiego 27, PL-50370 Wrocław, Poland; ‡ Department of Chemistry, Indian Institute of Technology Bhilai, Sejbahar, Raipur, Chhattisgarh 492015, India

## Abstract

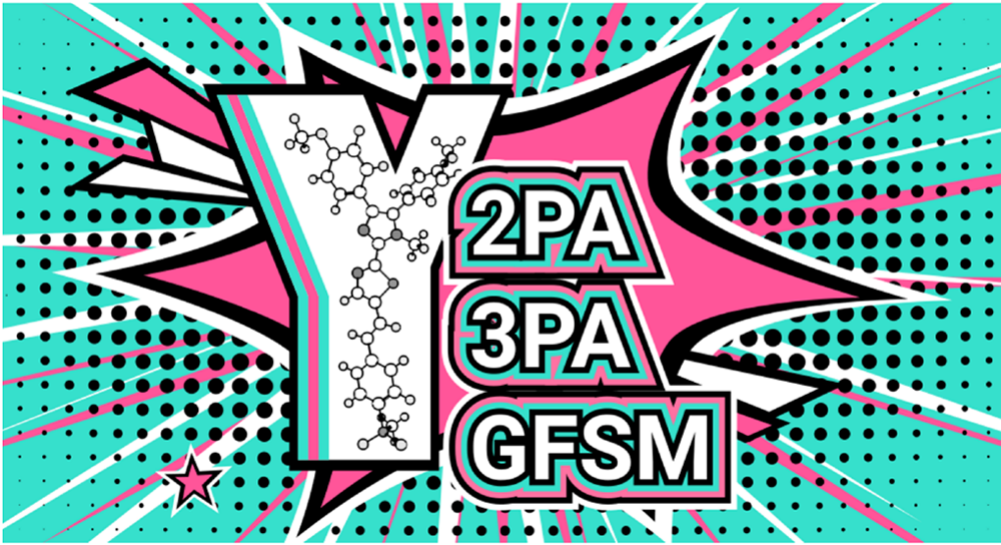

The molecular origin of two- (2PA) and three-photon absorption (3PA) activity in three experimentally studied chromophores, prototypical dipolar systems, is investigated. To that end, a generalized few-state model (GFSM) formula is derived for the 3PA transition strength for nonhermitian theories and employed at the coupled-cluster level of theory. Using various computational techniques such as molecular dynamics, linear and quadratic response theories, and GFSM, an in-depth analysis of various optical channels involved in 2PA and 3PA processes is presented. It is found that the four-state model involving the second and third excited singlet states as intermediates is the smallest model among all considered few-state approximations that produces 2PA and 3PA transition strengths (for S_0_ → S_1_ transition) close to the reference results. By analyzing various optical channels appearing in these models and involved in studied multiphoton processes, we found that the 2PA and 3PA activities in all the three chromophores are dominated and hence controlled by the dipole moment of the final excited state. The similar origins of the 2PA and the 3PA in these prototypical dipolar chromophores suggest transferability of structure–property relations from the 2PA to the 3PA domain.

## 1 Introduction

The invention of lasers in 1960^1^ initiated a series of discoveries of various nonlinear optical phenomena, eventually marking the birth of nonlinear optics that was theoretically predicted much earlier in 1930 by the Nobel Laureate Maria Göppert-Mayer.^2^ One of the milestones in this area was the experimental confirmation of multiphoton absorption;^3^ in such process two or more photons are simultaneously absorbed to reach higher excited state. The lowest-order multiphoton process, namely, two-photon absorption (2PA), is nowadays in the limelight due to its diverse applications, such as photodynamic therapy,^4−6^ bioimaging,^7−11^ three-dimensional optical data storage,^12,13^ microfabrication,^14^ and two-photon lasing,^15^ to name a few. The two-photon microscope is now standard equipment in biology or neuroscience laboratories, providing high-resolution images at the cellular level. Nevertheless, it has some limitations; e.g., the maximum imaging depth of such a device is restricted by tissue scattering. Introduction of higher-order multiphoton absorption processes in imaging techniques has been adopted as the solution for elimination of background noises. The three-photon fluorescence microscopy, which utilizes three-photon absorption (3PA) as the excitation mechanism, has been demonstrated to be a powerful technique for imaging deeply in tissues.^16−19^ As lower-energy photons are less scattered and multiphoton excitation causes significant background suppression, three-photon microscope provides clear images of regions within tissue that are unreachable by its two-photon analogue. Xu et al. demonstrated an efficient, noninvasive three-photon microscope that, in contrast to a two-photon microscope, enabled in vivo visualization of subcortical structures within an intact mouse brain.^16^ The above-mentioned technological advances contributed to the increased interest in higher-order multiphoton absorption effects. The design of multiphoton absorbing materials has become an important challenge in the field of material sciences. The need for maximizing the 2PA strengths has prompted studies on structure–property relationships. Early reports in this field revealed great potential of organic, organometallic, and dendrimeric molecules of dipolar, quadrupolar, and octupolar structures as 2PA materials for varios applications.^20,21^ However, the focus has now shifted toward metal–organic frameworks, perovskites, and materials with reduced dimensionality (particularly 2D materials).^22−25^ Although 3PA-based imaging promises to be a robust competitor to known 2PA techniques, 3PA processes are still much less explored than 2PA phenomena, on both the experimental and theoretical side. It stems from the challenges of experimental measurements of multiphoton effects, as the probability of simultaneous absorption of three and more photons is much smaller than that in the 2PA process. Therefore, it is necessary to use laser sources providing ultrashort laser pulses. From the computational point of view, the crucial factor is the cost of calculating these processes which, in many cases, can be very prohibitive, especially when highly accurate electronic-structure methods are employed. The developments in experimental techniques and modern computing infrastructures make the studies on multiphoton absorption feasible nowadays. In this work, we contribute to these efforts and make an attempt to facilitate the development of structure–property rules allowing for rational design of multiphoton absorbing materials.

## 2 Theory and Computational Details

The analysis of “structure–multiphoton absorption” relationships will be performed in this work on the basis of the results of electronic-structure calculations and generalized few-state models (GFSM), which were successfully employed in the field of nonlinear optical activity (mainly two-photon absorption) of molecular materials.^26−32^ The underlying concept comes from the sum-over-states method, but the key difference is that only a limited number of intermediate states are included in GFSM. As a result, the cost of computations is greatly reduced. Careful selection of the essential states with largest contribution to the observed nonlinear optical response is necessary to obtain satisfactory results. The key advantage of GFSM formalism is that it allows one to express multiphoton absorption strengths (or other nonlinear optical properties^33^) in terms of electronic structure parameters, i.e., excitation energies, dipole moments, and transition moments. Hence, one can thoroughly investigate the nature of multiphoton responses. Moreover, this approach utilizes the concept of optical channels, defined as a specific transitions between two states, and their interference.^26−28,31^ On the basis of GFSM, one can evaluate the contribution of individual optical channels to the overall mutiphoton response of the considered chemical system. The GFSM expression to calculate the 2PA strength at the coupled-cluster level (note that due to nonhermitian structure of coupled-cluster theory left and right transition moments may differ) was derived previously^32^ and is given by
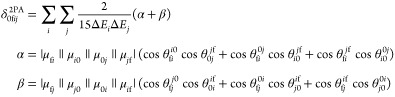
1where the subscripts distinguish between right (*j*0) and left moments (0*j*),  (ω_f_ represents the excitation energy for 0 → f transition) and θ_*pq*_^*rs*^ is the angle between the transition dipole moment vectors μ_*pq*_ and μ_*rs*_. In this work, we extended the GFSM for nonhermitian formulation of coupled-cluster response properties, thus allowing for analysis of the 3PA strength of studied molecules (the derivations can be found in the Supporting Information file):
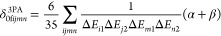
2
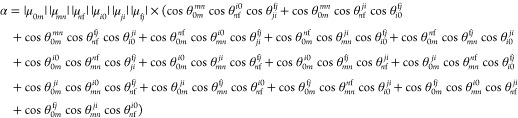

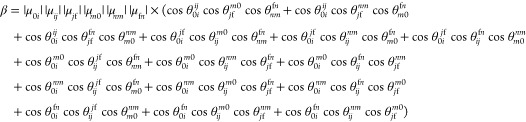
where Δ*E*_*i*1_ = ω_*i*_ – ω_f_/3 and Δ*E*_*j*2_ = ω_*j*_ – 2ω_f_/3. Any number of states (as indicated by *i*, *j*, *m*, *n*) can be chosen in eqs 1 and 2. For instance, in the two-state model (2SM) for the 3PA strength *i*, *j*, *m*, and *n* can be either the ground state 0 or the final excited state f. In what follows, the labeling NSM(*i*, ..., *j*, ...) will denote the *N*-state model with states *i*, ..., *j*, ... included as intermediate ones.

**Scheme 1:**
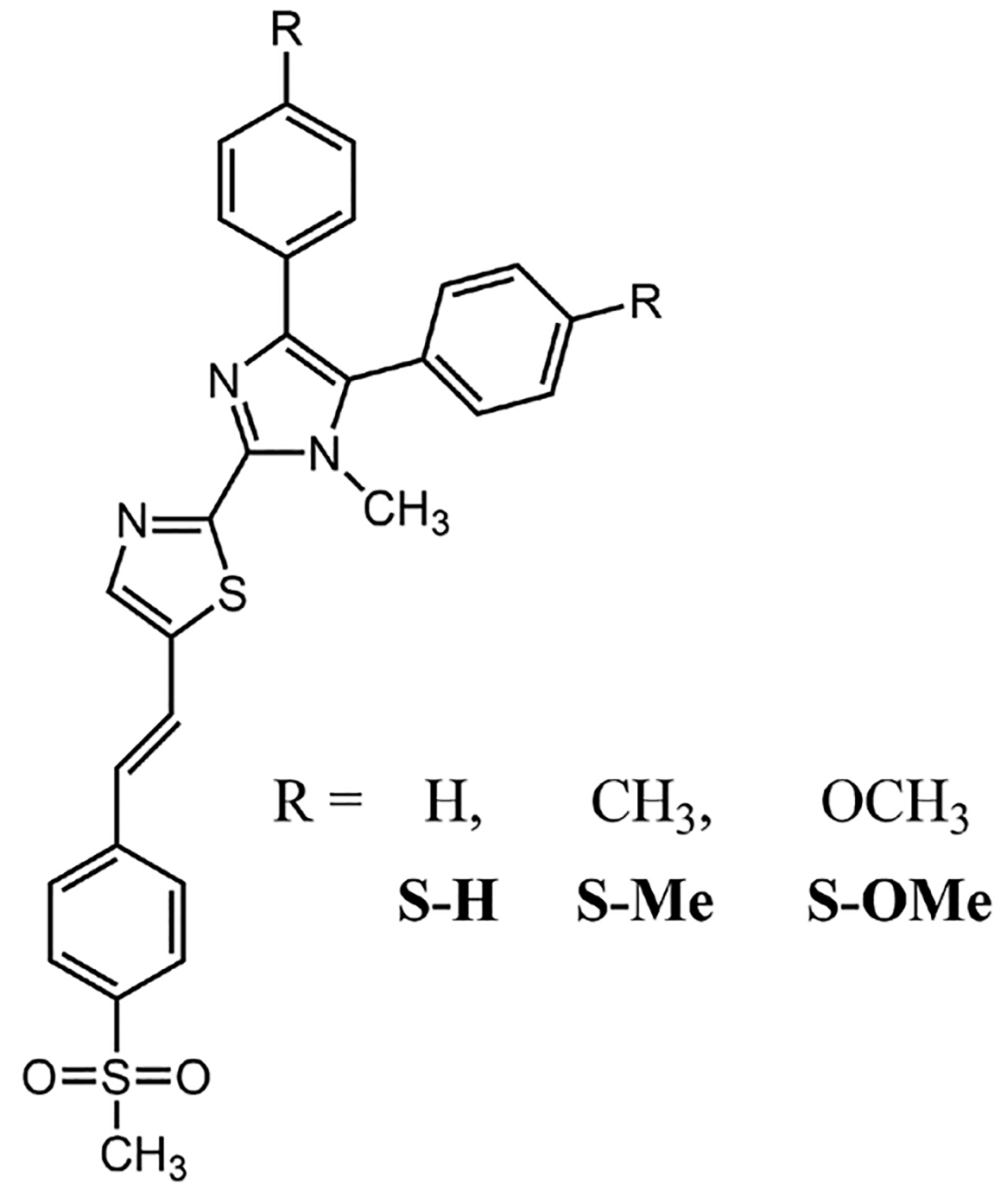
Structure of the Studied Compounds

In this work, we will perform analyses of the obtained GFSM formula by thorough comparison of two- and three-photon absorption properties of dipolar Y-shaped chromophores based on an imidazole–thiazole skeleton (see Scheme 1). There are two primary reasons behind selection of these systems for the present work: (a) experimental 2PA and 3PA cross sections were determined under same experimental conditions,^34^ and (b) there is a vast literature on the 2PA of dipolar systems, thus enabling comparative analysis of the 3PA activity more potent. It should be highlighted that the multibranched topology of studied compounds may indicate significant vibronic contributions to multiphoton absorption cross sections.^35^ However, this aspect is beyond the scope of the present study.

**Figure 1:**
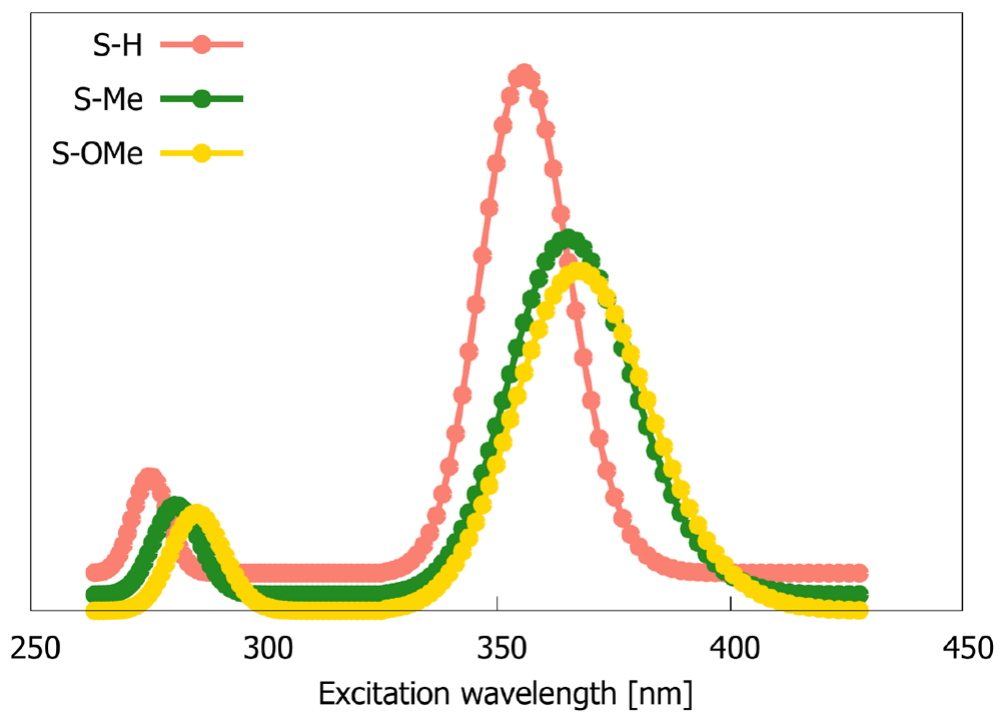
Simulated 1PA spectra of S–H, S–Me, and S–OMe molecules. Shown is the relative intensity.

The geometries of studied molecules (hereafter denoted as S–H, S–Me, S–OMe) were optimized at the B3LYP/6-31G(d,p)^36^ level of theory accounting for solvent effects (dimethyl sulfoxide solution) based on the Polarizable Continuum Model as implemented in the Gaussian 16 program.^37^ The stationary points on the potential energy hypersurface were confirmed to be minima by the evaluation of the Hessian. Subsequently, rigid-body molecular dynamic simulations were performed using NAMD program.^38^ In these simulations, the chromophore geometry was kept frozen to avoid its misrepresentation by a classical force field, as it is well-known that nonlinear optical properties are crucially dependent on geometrical parameters of π-conjugated moieties. A total of 100 snapshots were taken from the resulting trajectory for electronic-structure calculations at the RI-CC2/cc-pVDZ level of theory with the aid of the TURBOMOLE 7.3 program.^39,40^ In more detail, we employed the electrostatic embedding approximation; i.e., solvent molecules surrounding a chromophore were represented by point charges. For further analysis of the two- and three-photon absorption processes in terms of the GFSM based on the RI-CC2 method, we selected, separately for S–H, S–Me, and S–OMe, the chromophore-solvent snapshot with the S_0_ → S_1_ excitation energy closest to the arithmetic mean value of energy in the considered set of snapshots. More details can be found in the Supporting Information file.

## 3 Results and Discussion

**Figure 2:**
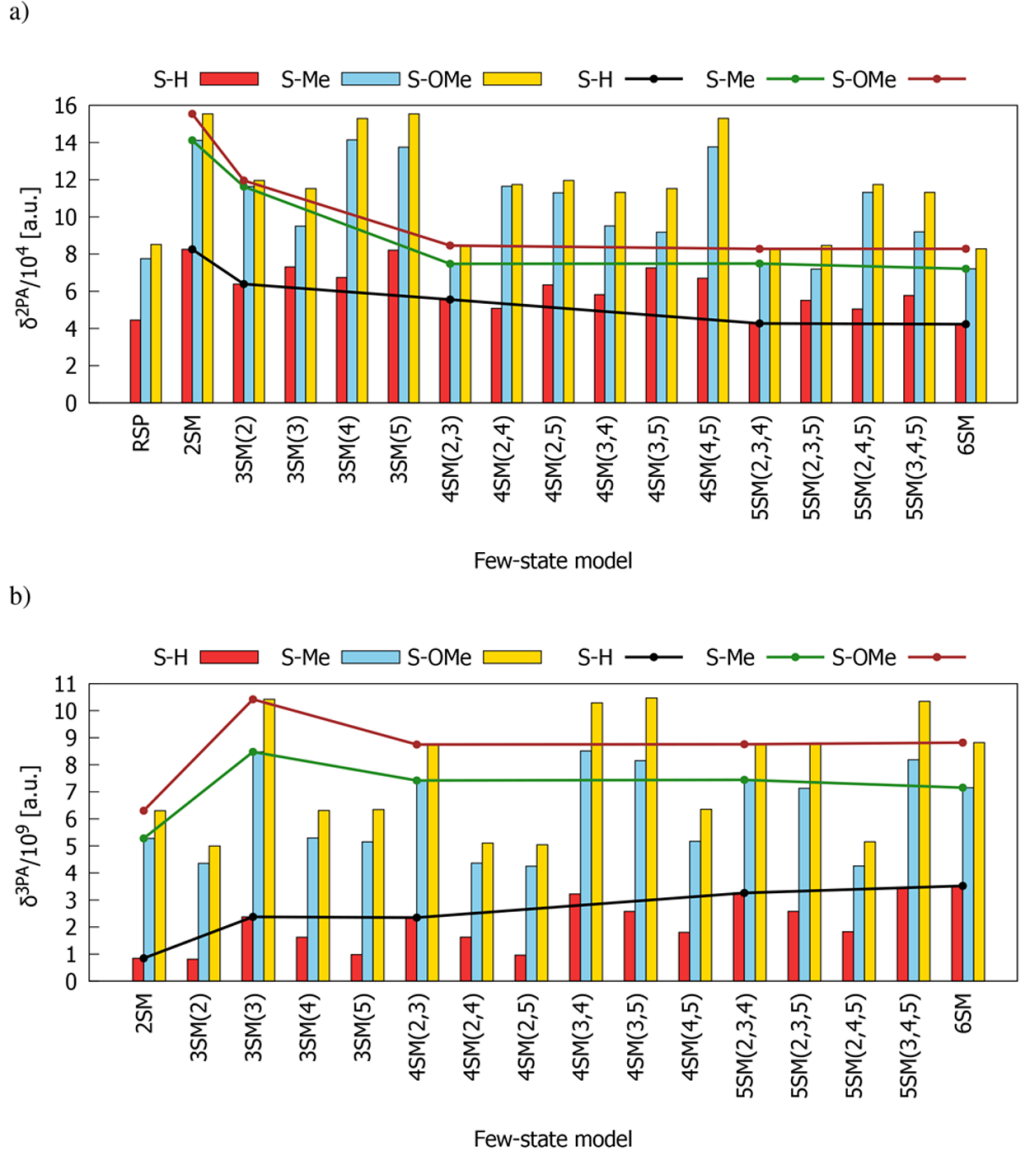
Comparison of response theory and few-state model S_0_ → S_1_ transition strengths for (a) 2PA and (b) 3PA processes. Solid lines show δ^2PA^ and δ^3PA^ convergence with respect to the number of electronic states.

We will start the discussion with the analysis of the results obtained using response theory (RSP) and various GFSM variants. Figures 1 and S1 show, respectively, the simulated and experimental electronic one-photon absorption spectra of S–H, S–Me, and S–OMe, revealing (a) the presence of strong low-lying ππ*-type excited state, typical for dipolar push–pull chromophores, and (b) satisfactory predictions of spectral shifts by the RI-CC2 method. The experimentally determined absorption band broadenings are underestimated in rigid-body MD simulations, as the vibrational fine structure is neglected.^41−45^ The SI file contains the analysis of electronic excitations to lowest-lying states based on the calculations performed using time-dependent density functional theory. Figure 2 presents the values of two- and three-photon absorption strength of S–H, S–Me, and S–OMe molecules computed for the S_0_ → S_1_ transition (hearafter intermediate states involved are given in brackets after abbreviation of the few-state model used). As it is seen, RSP and all few-state models (FSMs) predict an increasing trend of δ^2PA^ and δ^3PA^ on passing from S–H, through S–Me, to S–OMe. Most of the considered FSMs overestimate δ^2PA^ with respect to the reference RSP data. In turn, if we assume 6SM results as a reference for 3PA investigations, we can see that δ^3PA^ values are underestimated by most of the other few-state approximations. 2PA and 3PA strengths predicted for S–Me and S–OMe are significantly larger than the corresponding values obtained for S–H. As expected, the presence of strong electron-donating groups is the reason for the increased two- and three-photon responses of the studied compounds. Solid lines on Figure 2a,b illustrate the convergence of δ^2PA^ and δ^3PA^ with respect to the number of electronic excited states. Based on that, it is clear that 4SM(2,3) is the smallest model (i.e., a model that includes the smallest number of states) among all that gives satisfactory results (i.e., close to those predicted by RSP and 6SM), because adding more states to the analysis does not cause significant changes in δ^2PA^ and δ^3PA^. It is worth mentioning that 2SM, which is very often used in the analysis of two-photon responses, is not sufficient to properly describe the 2PA strength of the molecules considered herein, as it provides results that are significantly overestimated in comparison to RSP and 6SM outcomes. In the case of the S_0_ → S_2_ transition, the RSP calculations show that δ^2PA^ of S–H prevails over the results obtained for S–Me and S–OMe (see Figure S26 in the SI). δ^2PA^ of the S–Me molecule is significantly smaller than δ^2PA^ of the other two molecules. For studied compounds, all FSMs that include S_1_ state predict the same behavior of two-photon response as RSP. Furthermore, they all give similar δ^2PA^ values that come close to those predicted by RSP computations. Therefore, one can select 3SM(1) with confidence as the smallest model that gives satisfactory results. More significant differences between the results provided by FSMs are observed for the 3PA (S_0_ → S_2_) and it is obvious that 3SM(1) is not sufficient to properly describe δ^3PA^. One should choose at least 4SM(1,3) to obtain δ^3PA^ values close to these based on 6SM. All FSMs that include the S_1_ excited state demonstrate, similarly to the case of the S_0_ → S_1_ transition, an increase of δ^3PA^ across the series of studied compounds.

**Figure 3:**
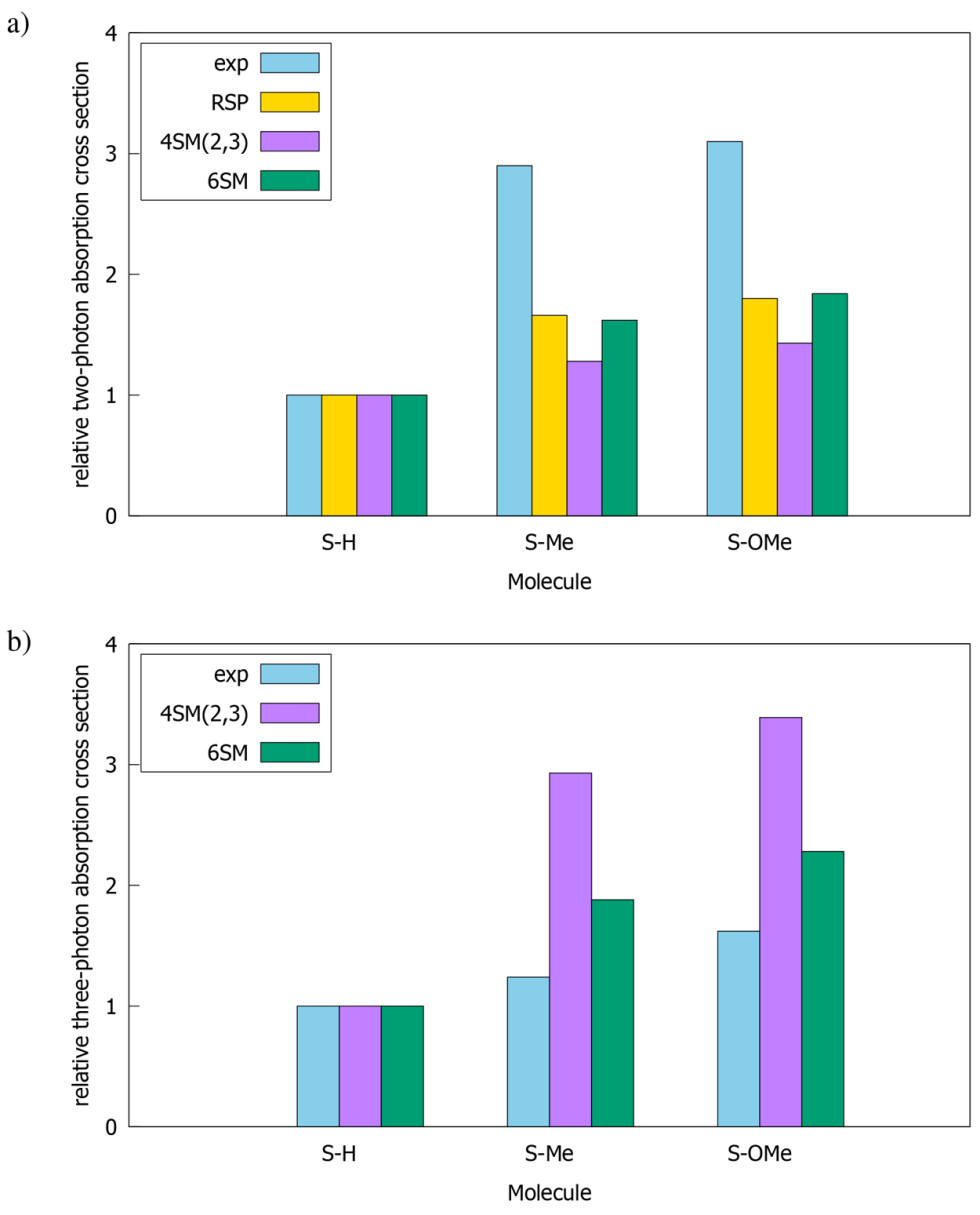
Comparison of calculated and experimental S_0_ → S_1_ cross sections  for (a) 2PA and (b) 3PA processes. See text for details.

The experimental measurements, performed previously at 750 and 1400 nm (therefore, they concern the S_0_ → S_1_ transition) by Mendonça et al.^34^ with the aid of open aperture Z-scan technique, show that 2PA and 3PA cross sections (σ_exp_^2PA^ and σ_exp_^3PA^, respectively) increase on passing from S–H, through S–Me, to S–OMe (see Table 1 in ref (34)). σ_exp_^2PA^ of S–Me is almost 3-fold larger than that determined for S–H, whereas the growth of σ_exp_^2PA^ between S–Me and S–OMe compounds is very small. On the other hand, the increase of σ_exp_^3PA^ is much steadier. The comparison of experimental and theoretical findings for S_0_ → S_1_ transition are given in Figure 3. To that end, we calculated the relative two- and three-photon absorption cross sections using the formula: σ_rel_^*n*PA^ = , where *ℏω* is the photon energy and *n* is equal to 2 (2PA) or 3 (3PA). For comparison, we only used results obtained from RSP and 4SM(2,3) calculations (as it was previously selected as the smallest model that provides reliable predictions) and 6SM (that gives the most accurate values among all considered FSMs). The data presented in Figure 3 show that although RSP, 4SM(2,3), and 6SM correctly predict the growing trend of 2PA and 3PA activity, the values of σ_rel_^2PA^ and σ_rel_^3PA^ deviate from the results of experimental measurements.

**Figure 4:**
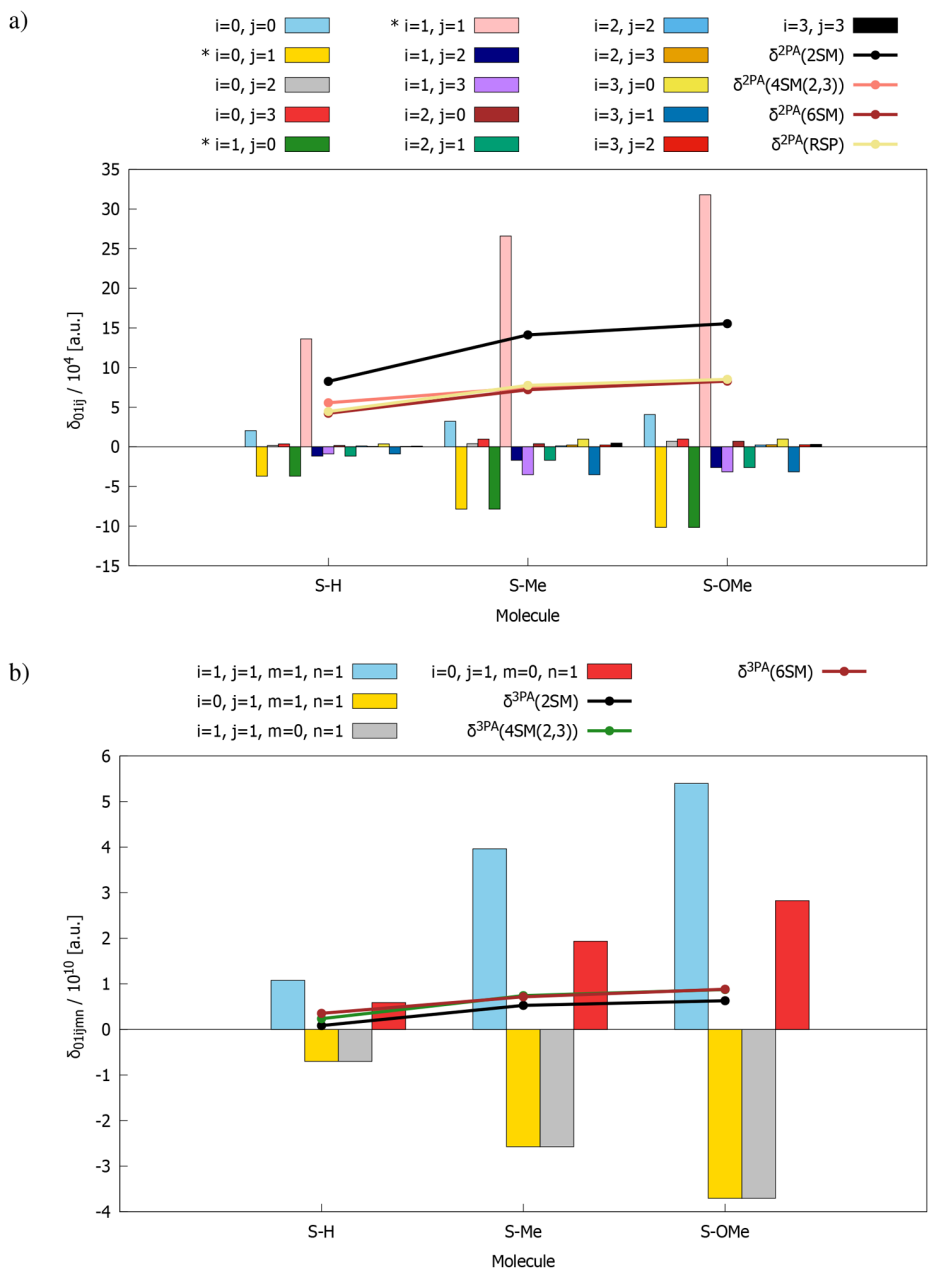
(a) Components of δ^2PA^(S_0_ → S_1_) within 4SM(2,3). (* denotes the highest values of δ_01*ij*_ among all components of δ^2PA^(S_0_ → S_1_) within 2SM, 3SM, and 4SM models. (b) Four highest δ_01*ijmn*_ values among all components of δ^3PA^(S_0_ → S_1_) within 2SM, 3SM, and 4SM models.

A better understanding of the origins of the 2PA and the 3PA of the studied molecules is obtained by analyzing the individual terms contributing to δ^2PA^ and δ^3PA^ within previously selected FSMs. It is clear from Figure 4a that for all molecules, in the case of the S_0_ → S_1_ transition, δ^2PA^(4SM(2,3)) is largely dominated by the δ_0111_ term (note that Cartesian components of dipole moments in the S_0_ and S_1_ states are given in Table S6 in the SI file). Moreover, the behavior of this component follows the growing trend observed for the total δ^2PA^ obtained using RSP and all FSMs. Note also that δ_0101_ and δ_0110_, although more than 3 times smaller than δ_0111_, contribute substantially to δ^2PA^(4SM(2,3)) in contrast to other terms. Due to the negative sign, δ_0101_ and δ_0110_ decrease the total value of δ^2PA^(4SM(2,3)). Figure 4b presents four terms, i.e., δ_011111_, δ_010111_, δ_011101_, and δ_010101_, that contribute the most to δ^3PA^ (for S_0_ → S_1_ transition) within 4SM(2,3) as well as 2SM and all three- and four-state models (see Figure S28 in the SI where we demonstrated all components of δ^3PA^ within one selected 3SM(3) model in descending order). As it is seen, all four components have quite significant contributions; however, δ_011111_ prevails. The absolute values of these components increase across the series of studied molecules. Because of the sign difference, these four terms cancel each other out to a large extent, and as a result, the values of total δ^3PA^ within 4SM(2,3) and other FSMs are small compared to δ_011111_, δ_010111_, δ_011101_, and δ_010101_, especially in the case of S–Me and S–OMe. As for the S_0_ → S_1_ transition, there are three terms with significant contribution to δ^2PA^ obtained for S_0_ → S_2_ within 3SM(1); i.e., δ_0211_, δ_0212_, δ_0221_, and δ_0211_ clearly dominate (see Figure S27 in the SI). In contrast to δ_0212_ and δ_0221_ components, δ_0211_ shows nonmonotonic behavior on passing from one molecule to another (the same as total δ^2PA^(3SM(1)), δ^2PA^(6SM), and δ^2PA^(RSP)). Also in the case of δ^3PA^(S_0_ → S_2_) one can select at least three terms within 4SM(1,3), i.e., δ_021111_, δ_020111_, and δ_021101_, with substantial contributions (see Figure S29 in the SI where we demonstrated all components of δ^3PA^ within one selected 3SM(1) model in descending order). Nevertheless, δ_021111_ dominates over all of them and exhibits a growth, which is in line with the behavior of total δ^3PA^(4SM(1,3)) and δ^3PA^(6SM).

The δ_0111_, δ_0211_, δ_011111_, and δ_021111_ components, contributing the most to δ^2PA^ and δ^3PA^ within few-state approximation, are directly related to electronic structure parameters according to the following formulas:
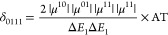

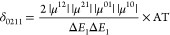

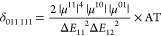

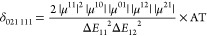
where AT stands for the angular part omitted for brevity. Figure S30 presents the breakdown of δ_0111_, δ_0211_, δ_011111_, and δ_021111_ into “energy” and “dipole” terms. The most significant changes are observed for the |μ^11^||μ^11^| component. There is an increase of its value by around 40 au between S–H and S–OMe. The other two “dipole” terms undergo much smaller variations. Similarly, the changes in “energy” components on passing from one molecule to another are very minor. Therefore, we can conclude that in the case of the S_0_ → S_1_ transition the dipole moment of excited state S_1_ determines the behavior of δ^2PA^ and δ^3PA^. The same is true for three-photon excitation to S_2_ state. However, the 2PA response of the studied molecules for S_0_ → S_2_ is mainly governed by variations in transition moments between S_1_ and S_2_ states.

## 4 Summary

In summary, using various techniques such as molecular dynamics, linear and quadratic response theory, and generalized few-state models (GFSM) at the ab initio RI-CC2 level of theory, we studied the two- and three-photon excitations to the first and the second excited singlet states in three experimentally described chromophores, representing prototypical dipolar systems. To that end, a novel nonhermitian GFSM formula for three-photon absorption strengths is derived and employed at the coupled-cluster level. A four-state model involving the second and third excited singlet states as intermediates is found to be the smallest model among all considered few-state approximations to produce 2PA and 3PA transition strengths (for S_0_ → S_1_ transition) close to the reference results (i.e., obtained from response theories or on the basis of 6SM). By analyzing various optical channels appearing in these models and involved in 2PA and 3PA processes, we found that the said two- and three-photon activities in all the three chromophores are dominated and hence controlled by the dipole moment of the final excited state. The similar origins of the 2PA and the 3PA in these prototypical dipolar chromophores suggest transferability of structure–property relations from the 2PA to the 3PA domain.

## Supporting Information Available

Derivation of generalized few-state model for three-photon absorption, description of molecular dynamics simulations, results of electronic-structure calculations using time-dependent density functional and coupled-cluster theory, decompositions of two- and three-photon transition strengths using few-state models; figures of PA spectra, orbitals involved in transitions, excitation energy distributions, comparison of response theory and few-state model results, δ components, energy and dipole components, and molecular orientation; tables of charge transfer parameters, excitation energies and wavelengths, oscillator and transition strengths, and orbitals, and dipole moments (PDF)
