# Supplementary material for: Much of a Muchness: On the Origins of Two- and Three-Photon Absorption Activity of Dipolar Y-Shaped Chromophores
Source: J Phys Chem A. 2022 Jan 27;126(5):752–9. doi: 10.1021/acs.jpca.1c10098 (PMC8842251; doi:10.1021/acs.jpca.1c10098)
Supplement: Supplementary file 1 — jp1c10098_si_001.pdf [file jp1c10098_si_001.pdf]

Supporting Information
Much of a Muchness: On the Origins of Two- and Three-Photon Absorption Activity of Dipolar Y-Shaped Chromophores

Marta Chołuj,[*,†] Rojalini Behera,[‡] Elizaveta F. Petrusevich,[†] Wojciech Bartkowiak,[†] Md. Mehboob Alam,[*,‡] and Robert Zaleśny[*,†]

†*Faculty of Chemistry, Wrocław University of Science and Technology, Wyb. Wyspiańskiego 27, PL–50370 Wrocław, Poland*

‡*Department of Chemistry, Indian Institute of Technology Bhilai, Sejbahar, Raipur, Chhattisgarh – 492015, India*

E-mail: marta.choluj@pwr.edu.pl; mehboob@iitbhilai.ac.in; robert.zalesny@pwr.edu.pl

Derivation of generalized few state formula for three-photon absorption

In the case of a single beam of linearly polarized light the three-photon absorption strength within the framework of coupled-cluster theory is given by[1]

$$\delta_{0f}^{3PA} = \frac{1}{35} \sum_{abc} \left(2M_{0\leftarrow f}^{abc} M_{f\leftarrow 0}^{abc} + 3M_{0\leftarrow f}^{aab} M_{f\leftarrow 0}^{bcc} \right), \qquad a, b, c \in \{x, y, z\}. \tag{1}$$

$M_{f\leftarrow 0}^{abc}$ and $M_{0\leftarrow f}^{abc}$ denote the abc-th component of right and left three-photon transition moment, respectively, and are given by[2]

$$M_{f\leftarrow 0}^{abc} = M_R^{abc} = \hat{P}^{abc} \sum_{ij} \frac{\mu_{i0}^a \mu_{ji}^b \mu_{fj}^c}{\Delta E_{i1} \Delta E_{j2}} \tag{2}$$

$$M_{0\leftarrow f}^{abc} = M_L^{abc} = \hat{P}^{abc} \sum_{ij} \frac{\mu_{0i}^a \mu_{ij}^b \mu_{jf}^c}{\Delta E_{i1} \Delta E_{j2}} \tag{3}$$

where $\hat{P}^{abc}$ is the permutation operator, $\Delta E_{i1} = \omega_i - \omega_f/3$, $\Delta E_{j2} = \omega_j - 2\omega_f/3$ (ω_i represents the excitation energy for $0 \rightarrow i$ transition) and $\mu_{0i}^a = \langle 0|\mu^a|i\rangle$ is the transition dipole moment between states 0 and i. The summations in Eqs. 2 and 3 run over all electronic states. Henceforth we will use the notation $a_{0i} = \mu_{0i}^a$, $b_{ij} = \mu_{ij}^b$, $c_{jf} = \mu_{jf}^c$, so that

$$M_{f\leftarrow 0}^{abc} = \sum_{ij} \frac{a_{i0}b_{ji}c_{fj} + a_{i0}c_{ji}b_{fj} + b_{i0}a_{ji}c_{fj} + b_{i0}c_{ji}a_{fj} + c_{i0}a_{ji}b_{fj} + c_{i0}b_{ji}a_{fj}}{\Delta E_{i1} \Delta E_{j2}}. \tag{4}$$

By expanding Eq. 1 (using notation from Eqs. 2 and 3) we get

$$
\begin{aligned}
\delta_{0f}^{3PA} &= \frac{1}{35}\sum_{abc}\left(2M_{0\leftarrow f}^{abc}M_{f\leftarrow 0}^{abc} + 3M_{0\leftarrow f}^{aab}M_{f\leftarrow 0}^{bcc}\right) \\
&= \frac{1}{35}[5M_L^{xxx}M_R^{xxx} + 5M_L^{yyy}M_R^{yyy} + 5M_L^{zzz}M_R^{zzz} \\
&+ 9M_L^{xxy}M_R^{xxy} + 9M_L^{xxz}M_R^{xxz} + 9M_L^{yyx}M_R^{yyx} + 9M_L^{yyz}M_R^{yyz} + 9M_L^{zzx}M_R^{zzx} + 9M_L^{zzy}M_R^{zzy} \\
&+ 3M_L^{xxx}M_R^{xyy} + 3M_L^{xxx}M_R^{xzz} + 3M_L^{yyy}M_R^{yxx} + 3M_L^{yyy}M_R^{yzz} + 3M_L^{zzz}M_R^{zxx} + 3M_L^{zzz}M_R^{zyy} \\
&+ 3M_L^{xxy}M_R^{yyy} + 3M_L^{xxy}M_R^{yzz} + 3M_L^{yyx}M_R^{xxx} + 3M_L^{yyx}M_R^{xzz} + 3M_L^{zzx}M_R^{xxx} + 3M_L^{zzx}M_R^{xyy} \\
&+ 3M_L^{xxz}M_R^{zyy} + 3M_L^{xxz}M_R^{zzz} + 3M_L^{yyz}M_R^{zxx} + 3M_L^{yyz}M_R^{zzz} + 3M_L^{zzy}M_R^{yxx} + 3M_L^{zzy}M_R^{yyy} \\
&+ 12M_L^{xyz}M_R^{xyz}]
\end{aligned} \tag{5}
$$

Let us look at the first term in Eq. 5

$$
\begin{aligned}
\delta_{0fijmn}^{3PA} &= \frac{1}{35}[5M_L^{xxx}M_R^{xxx} + ...] = \frac{1}{35}[5\cdot\sum_{ij}\frac{6x_{0i}x_{ij}x_{jf}}{\Delta E_{i1}\Delta E_{j2}}\sum_{mn}\frac{6x_{m0}x_{nm}x_{fn}}{\Delta E_{m1}\Delta E_{n2}} + ...] \\
&= \frac{1}{35}[5\cdot\sum_{ijmn}\frac{6x_{0i}x_{ij}x_{jf}}{\Delta E_{i1}\Delta E_{j2}}\cdot\frac{6x_{m0}x_{nm}x_{fn}}{\Delta E_{m1}\Delta E_{n2}} + ...] \\
&= \frac{1}{35}[5\cdot\frac{1}{2}\sum_{ijmn}\left(\frac{6x_{0i}x_{ij}x_{jf}}{\Delta E_{i1}\Delta E_{j2}} + \frac{6x_{0m}x_{mn}x_{nf}}{\Delta E_{m1}\Delta E_{n2}}\right)\left(\frac{6x_{i0}x_{ji}x_{fj}}{\Delta E_{i1}\Delta E_{j2}} + \frac{6x_{m0}x_{nm}x_{fn}}{\Delta E_{m1}\Delta E_{n2}}\right) + ...]
\end{aligned} \tag{6}
$$

Hence, if we only consider terms where $i, j \neq m, n$ we get

$$
\delta_{0fijmn}^{3PA} = \frac{1}{2}\cdot\frac{1}{35}\sum_{ijmn}\frac{1}{\Delta E_{i1}\Delta E_{j2}\Delta E_{m1}\Delta E_{n2}}\left(180x_{0i}x_{ij}x_{jf}x_{m0}x_{nm}x_{fn} + 180x_{0m}x_{mn}x_{nf}x_{i0}x_{ji}x_{fj} + ...\right) \tag{7}
$$

After doing the analogous transformations as in Eq. 6 for each terms in Eq. 5 we will get the same set of terms for $0i, ij, jf, m0, nm, fn$ and $0m, mn, nf, i0, ji, fj$ indices

$$\delta^{3PA}_{0fijmn} = \frac{1}{2} \cdot \frac{1}{35} \sum_{ijmn} \frac{1}{\Delta E_{i1} \Delta E_{j2} \Delta E_{m1} \Delta E_{n2}} \Big(180xxxxxx + 180yyyyyy + 180zzzzzz$$

$$+ 36xxyxxy + 36xxyxyx + 36xxyyxx + 36xyxxxy + 36xyxxyx + 36xyxyxx$$

$$+ 36yxxxxy + 36yxxxyx + 36yxxyxx$$

$$+ 36xxzxxz + 36xxzxzx + 36xxzzxx + 36xzxxxz + 36xzxxzx + 36xzxzxx$$

$$+ 36zxxxxz + 36zxxxzx + 36zxxzxx$$

$$+ 36yyxyyx + 36yyxyxy + 36yyxxyy + 36yxyyyx + 36yxyyxy + 36yxyxyy$$

$$+ 36xyyyyx + 36xyyyxy + 36xyyxyy$$

$$+ 36yyzyyz + 36yyzyzy + 36yyzzyy + 36yzyyyz + 36yzyyzy + 36yzyzyy$$

$$+ 36zyyyyz + 36zyyyzy + 36zyyzyy$$

$$+ 36zzxzzx + 36zzxzxz + 36zzxxzz + 36zxzzzx + 36zxzzxz + 36zxzxzz$$

$$+ 36xzzzzx + 36xzzzxz + 36xzzxzz$$

$$+ 36zzyzzy + 36zzyzyz + 36zzyyzz + 36zyzzzy + 36zyzzyz + 36zyzyzz$$

$$+ 36yzzzzy + 36yzzzyz + 36yzzyzz$$

$$+ 36xxxxyy + 36xxxyxy + 36xxxyyx + 36xxxxzz + 36xxxzxz + 36xxxzzx$$

$$+ 36yyyyxx + 36yyyxyx + 36yyyxxy + 36yyyyzz + 36yyyzyz + 36yyyzzy$$

$$+ 36zzzzxx + 36zzzxzx + 36zzzxxz + 36zzzzyy + 36zzzyzy + 36zzzyyz$$

$$+ 12xxyyzz + 12xxyzyz + 12xxyzzy + 12xyxyzz + 12xyxzyz + 12xyxzzy$$

$$+ 12yxxyzz + 12yxxzyz + 12yxxzzy$$

$$+ 12yyxxzz + 12yyxzxz + 12yyxzzx + 12yxyxzz + 12yxyzxz + 12yxyzzx$$

$$+ 12xyyxzz + 12xyyzxz + 12xyyzzx$$

$$+ 12zzxxyy + 12zzxyxy + 12zzxyyx + 12zxzxyy + 12zxzyxy + 12zxzyyx$$

$$+ 12xzzzxyy + 12xzzzyxy + 12xzzzyyx$$

$$+ 12xxzzzyy + 12xxzzyzy + 12xxzzyyz + 12xzxzzyy + 12xzzxyzy + 12xzzxyyz$$

$$+ 12zxxzzyy + 12zxxyzy + 12zxxyyz$$

$$+ 12yyzzzxx + 12yyzzxzx + 12yyzxxz + 12yzyzzxx + 12yzyxzx + 12yzyxxz$$

$$+ 12zyyzzxx + 12zyyxzx + 12zyyxxz$$

$$+ 12zzyyxx + 12zzyxyx + 12zzyxxy + 12zyzyxx + 12zyzxyx + 12zyzxxy$$

$$+ 12yzzyxx + 12yzzxyx + 12yzzxxy$$

$$+ 36xxyyyy + 36xyxyyy + 36yxxyyy + 36yyxxxx + 36yxyxxx + 36xyyxxx$$

$$+ 36zzxxxx + 36zxzxxx + 36xzzxxx + 36xxzzzz + 36xzxzzz + 36zxxzzz$$

$$+ 36yyzzzz + 36yzyzzz + 36zyyzzz + 36zzyyyy + 36zyzyyy + 36yzzyyy$$

$$+ 12xyzxyz + 12xyzxzy + 12xyzyxz + 12xyzyzx + 12xyzzxy + 12xyzzyx$$

$$+ 12xzyxyz + 12xzyxzy + 12xzyyxz + 12xzyyzx + 12xzyzxy + 12xzyzyx$$

$$+ 12yxzxyz + 12yxzxzy + 12yxzyxz + 12yxzyzx + 12yxzzxy + 12yxzzyx$$

$$+ 12yzxxyz + 12yzxxzy + 12yzxyxz + 12yzxyzx + 12yzxzxy + 12yzxzyx$$

$$+ 12zxyxyz + 12zxyxzy + 12zxyyxz + 12zxyyzx + 12zxyzxy + 12zxyzyx$$

$$+ 12zyxxyz + 12zyxxzy + 12zyxyxz + 12zyxyzx + 12zyxzxy + 12zyxzyx$$

$$+ \text{the same set of terms for } 0m, mn, nf, i0, ji, fj\Big)$$

In the above equation we omitted the indices for each $xyzxyz$ term as they have always the same order, i.e. $0i, ij, jf, mo, nm, fn$. Following derivations presented previously,[1] separately for indices $0i, ij, jf, m0, nm, fn$ and $0m, mn, nf, i0, ji, fj$ we obtain

$$\delta^{3PA}_{0fijmn} = \frac{6}{35} \sum_{ijmn} \frac{1}{\Delta E_{i1} \Delta E_{j2} \Delta E_{m1} \Delta E_{n2}} (\alpha + \beta) \tag{8}$$

$$\alpha = |\mu_{0m}||\mu_{mn}||\mu_{nf}||\mu_{i0}||\mu_{ji}||\mu_{fj}|$$
$$\times (\cos\theta_{0m}^{mn}\cos\theta_{nf}^{i0}\cos\theta_{ji}^{fj} + \cos\theta_{0m}^{mn}\cos\theta_{nf}^{ji}\cos\theta_{i0}^{fj} + \cos\theta_{0m}^{mn}\cos\theta_{nf}^{fj}\cos\theta_{i0}^{ji}$$
$$+ \cos\theta_{0m}^{nf}\cos\theta_{mn}^{i0}\cos\theta_{ji}^{fj} + \cos\theta_{0m}^{nf}\cos\theta_{mn}^{ji}\cos\theta_{i0}^{fj} + \cos\theta_{0m}^{nf}\cos\theta_{mn}^{fj}\cos\theta_{i0}^{ji}$$
$$+ \cos\theta_{0m}^{i0}\cos\theta_{mn}^{nf}\cos\theta_{ji}^{fj} + \cos\theta_{0m}^{i0}\cos\theta_{mn}^{ji}\cos\theta_{nf}^{fj} + \cos\theta_{0m}^{i0}\cos\theta_{mn}^{fj}\cos\theta_{nf}^{ji}$$
$$+ \cos\theta_{0m}^{ji}\cos\theta_{mn}^{nf}\cos\theta_{i0}^{fj} + \cos\theta_{0m}^{ji}\cos\theta_{mn}^{i0}\cos\theta_{nf}^{fj} + \cos\theta_{0m}^{ji}\cos\theta_{mn}^{fj}\cos\theta_{nf}^{i0}$$
$$+ \cos\theta_{0m}^{fj}\cos\theta_{mn}^{nf}\cos\theta_{i0}^{ji} + \cos\theta_{0m}^{fj}\cos\theta_{mn}^{i0}\cos\theta_{nf}^{ji} + \cos\theta_{0m}^{fj}\cos\theta_{mn}^{ji}\cos\theta_{nf}^{i0})$$

$$\beta = |\mu_{0i}||\mu_{ij}||\mu_{jf}||\mu_{m0}||\mu_{nm}||\mu_{fn}|$$
$$\times (\cos\theta_{0i}^{ij}\cos\theta_{jf}^{m0}\cos\theta_{nm}^{fn} + \cos\theta_{0i}^{ij}\cos\theta_{jf}^{nm}\cos\theta_{m0}^{fn} + \cos\theta_{0i}^{ij}\cos\theta_{jf}^{fn}\cos\theta_{m0}^{nm}$$
$$+ \cos\theta_{0i}^{jf}\cos\theta_{ij}^{m0}\cos\theta_{nm}^{fn} + \cos\theta_{0i}^{jf}\cos\theta_{ij}^{nm}\cos\theta_{m0}^{fn} + \cos\theta_{0i}^{jf}\cos\theta_{ij}^{fn}\cos\theta_{m0}^{nm}$$
$$+ \cos\theta_{0i}^{m0}\cos\theta_{ij}^{jf}\cos\theta_{nm}^{fn} + \cos\theta_{0i}^{m0}\cos\theta_{ij}^{nm}\cos\theta_{jf}^{fn} + \cos\theta_{0i}^{m0}\cos\theta_{ij}^{fn}\cos\theta_{jf}^{nm}$$
$$+ \cos\theta_{0i}^{nm}\cos\theta_{ij}^{jf}\cos\theta_{m0}^{fn} + \cos\theta_{0i}^{nm}\cos\theta_{ij}^{m0}\cos\theta_{jf}^{fn} + \cos\theta_{0i}^{nm}\cos\theta_{ij}^{fn}\cos\theta_{jf}^{m0}$$
$$+ \cos\theta_{0i}^{fn}\cos\theta_{ij}^{jf}\cos\theta_{m0}^{nm} + \cos\theta_{0i}^{fn}\cos\theta_{ij}^{m0}\cos\theta_{jf}^{nm} + \cos\theta_{0i}^{fn}\cos\theta_{ij}^{nm}\cos\theta_{jf}^{m0})$$

where θ_{mn}^{0m} is the angle between the transition dipole moment vectors μ_{mn} and μ_{0m}.

Molecular dynamics simulations

Every studied molecule was solvated by adding, around optimized geometry of the studied dye (B3LYP/6-31G** level of theory, influence of solvent included by PCM), approximately 500 dimethyl sulfoxide (DMSO) molecules to form 40x40x40 Å box with the density close to the density of DMSO at room temperature (1.100 g/ml). VEGA ZZ software[3] was used in order to create DMSO box and to put dye inside. Total charge for each system was zero. Molecular dynamics simulations program NAMD[4] was used to perform simulations. The partial charges of three molecules and of DMSO molecules were obtained from B3LYP/6-31G** calculations at the equilibrium geometry using the CHELPG method. Lennard-Jones parameters taken from the CHARMM force field[5,6] were used to describe the dyes. For the description of DMSO force field parameters proposed by by Strader and Feller for condensed phases[7] were used. The system was minimized during 1000 steps followed by constant temperature NVT dynamics for 1 ns (1 step = 2 fs) at 300 K using a Langevin thermostat. MD simulations were carried out assuming fixed coordinates of dyes molecules (rigid-body MD). Periodic boundary conditions were applied. Once hydrogen bonds balance and energy equilibrium have been achieved, 100 snapshots (with a spacing step 1 ps) were collected from the resulting trajectory. Subsequently, electronic structure calculations were conducted for all 100 snapshots per system (300 snapshots in total) at RI-CC2/cc-pVDZ level of theory using TURBOMOLE-7.3 program.[8] Partial charges used for the description of solvent environment within electrostatic embedding (discrete solvent representation) were the same as within molecular dynamic simulations.

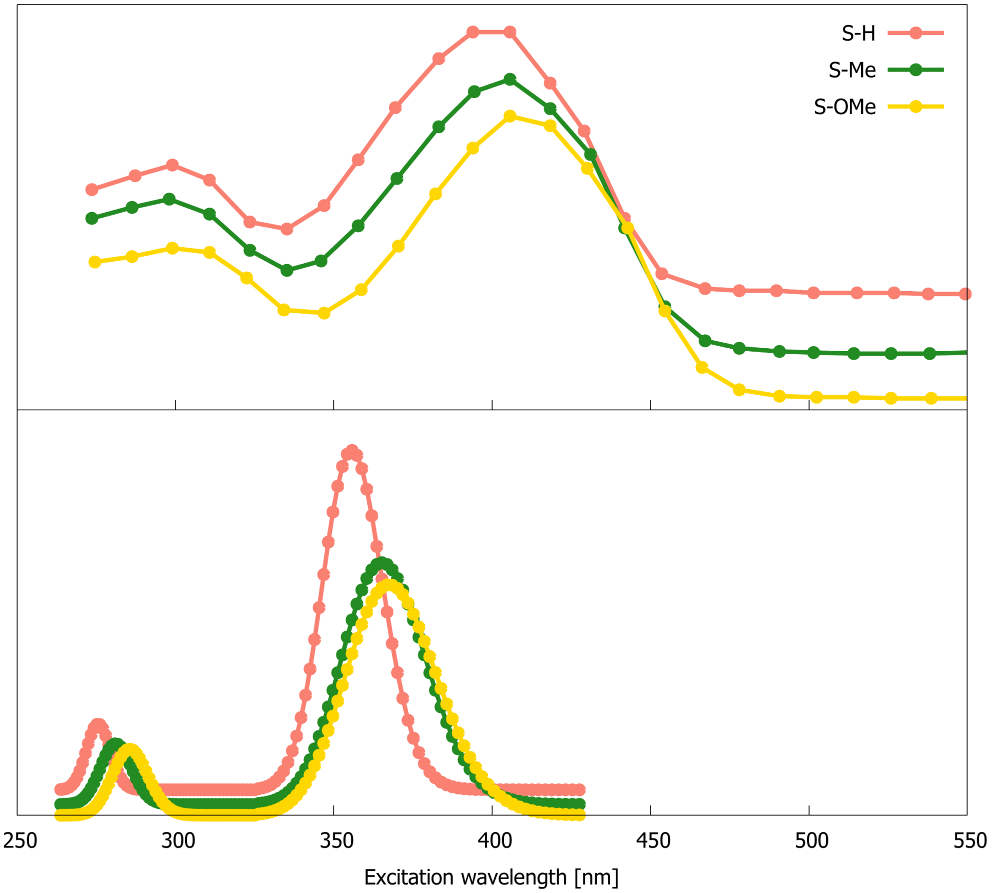

Figure S1: Experimental (top) and simulated (bottom) 1PA spectra of S-H, S-Me and S-OMe molecules. Shown is the relative intensity.

Analysis of electronic transitions within TD-DFT framework

Table S1: Charge transfer parameters D_{CT} (in Å) for $S_0 \rightarrow S_1$ transition obtained at CAM-B3LYP/aug-cc-pVDZ and M06-2X/aug-cc-pVDZ levels of theory.

	CAM-B3LYP	M06-2X
Molecule	D_{CT}	D_{CT}
S-H	2.798	3.355
S-CH_3	3.644	4.203
S-OCH_3	3.692	4.274

Table S2: Excitation energies (ΔE, in eV), excitation wavelengths (λ, in nm), oscillator strengths (f) and orbitals dominantly involved in transitions to the five lowest-energy singlet excited stated for structure S-H. The values were obtained at CAM-B3LYP/aug-cc-pVDZ and M06-2X/aug-cc-pVDZ levels of theory.

	CAM-B3LYP				M06-2X			
Transition	ΔE	λ	f	Orbitals involved	ΔE	λ	f	Orbitals involved
$S_0 \rightarrow S_1$	3.4341	361.04	2.0196	HOMO → LUMO	3.4454	359.86	2.0122	HOMO → LUMO
$S_0 \rightarrow S_2$	4.4463	278.85	0.2518	HOMO → LUMO+1	4.3997	281.80	0.2376	HOMO → LUMO+2
$S_0 \rightarrow S_3$	4.5457	272.75	0.0492	HOMO−1 → LUMO	4.5450	272.79	0.0617	HOMO−1 → LUMO
$S_0 \rightarrow S_4$	4.7154	262.93	0.1297	HOMO−1 → LUMO	4.6352	267.48	0.1587	HOMO−1 → LUMO
$S_0 \rightarrow S_5$	4.7668	260.10	0.0113	HOMO → LUMO+5	4.6904	264.33	0.0674	HOMO → LUMO+6

Table S3: Excitation energies (ΔE, in eV), excitation wavelengths (λ, in nm), oscillator strengths (f) and orbitals dominantly involved in transitions to the five lowest-energy singlet excited stated for structure S-CH3. The values were obtained at CAM-B3LYP/aug-cc-pVDZ and M06-2X/aug-cc-pVDZ levels of theory.

	CAM-B3LYP				M06-2X			
Transition	ΔE	λ	f	Orbitals involved	ΔE	λ	f	Orbitals involved
$S_0 \rightarrow S_1$	3.3684	368.08	1.9285	HOMO $\rightarrow$ LUMO	3.3641	368.55	1.8718	HOMO $\rightarrow$ LUMO
$S_0 \rightarrow S_2$	4.4023	281.64	0.1059	HOMO$-$1 $\rightarrow$ LUMO	4.3951	282.10	0.2073	HOMO$-$1 $\rightarrow$ LUMO
$S_0 \rightarrow S_3$	4.5769	270.89	0.2006	HOMO $\rightarrow$ LUMO+1	4.5075	275.06	0.2478	HOMO $\rightarrow$ LUMO+1
$S_0 \rightarrow S_4$	4.6714	265.41	0.3566	HOMO $\rightarrow$ LUMO+4	4.6248	268.09	0.1071	HOMO $\rightarrow$ LUMO+5
$S_0 \rightarrow S_5$	4.7656	260.16	0.0043	HOMO $\rightarrow$ LUMO+5	4.6514	266.55	0.2346	HOMO $\rightarrow$ LUMO+5

Table S4: Excitation energies (ΔE, in eV), excitation wavelengths (λ, in nm), oscillator strengths (f) and orbitals dominantly involved in transitions to the five lowest-energy singlet excited stated for structure S-OCH3. The values were obtained at CAM-B3LYP/aug-cc-pVDZ and M06-2X/aug-cc-pVDZ levels of theory.

	CAM-B3LYP				M06-2X			
Transition	ΔE	λ	f	Orbitals involved	ΔE	λ	f	Orbitals involved
$S_0 \to S_1$	3.3587	369.14	1.9821	HOMO $\to$ LUMO	3.3547	369.58	1.9256	HOMO $\to$ LUMO
$S_0 \to S_2$	4.3673	283.89	0.1643	HOMO$-$1 $\to$ LUMO	4.3483	285.13	0.2964	HOMO$-$1 $\to$ LUMO
$S_0 \to S_3$	4.5386	273.18	0.1080	HOMO $\to$ LUMO+1	4.4142	280.87	0.0053	HOMO $\to$ LUMO+2
$S_0 \to S_4$	4.6079	269.07	0.0700	HOMO $\to$ LUMO+2	4.4958	275.78	0.1569	HOMO $\to$ LUMO+1
$S_0 \to S_5$	4.6457	266.88	0.3842	HOMO $\to$ LUMO+6	4.6292	267.83	0.3594	HOMO $\to$ LUMO+6

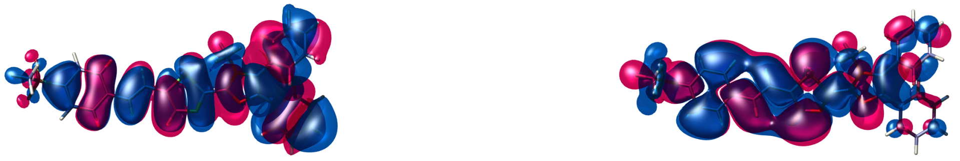

(a) HOMO (b) LUMO

Figure S2: Orbitals dominantly involved into $S_0 \rightarrow S_1$ transition for structure S-H, CAM-B3LYP/aug-cc-pVDZ level of theory.

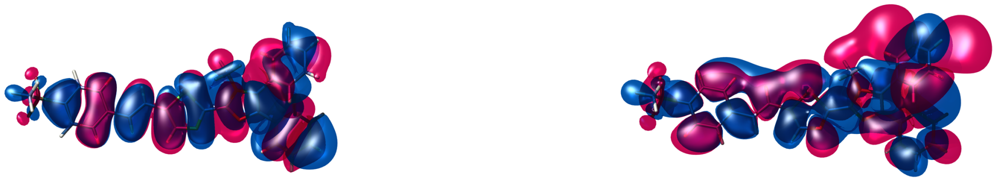

(a) HOMO (b) LUMO+1

Figure S3: Orbitals dominantly involved into $S_0 \rightarrow S_2$ transition for structure S-H, CAM-B3LYP/aug-cc-pVDZ level of theory.

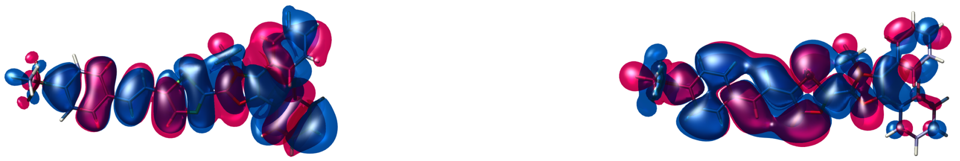

(a) HOMO (b) LUMO

Figure S4: Orbitals dominantly involved into $S_0 \rightarrow S_1$ transition for structure S-H, M06-2X/aug-cc-pVDZ level of theory.

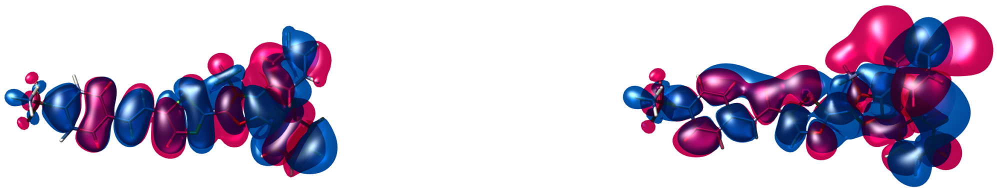

(a) HOMO (b) LUMO+2

Figure S5: Orbitals dominantly involved into $S_0 \rightarrow S_2$ transition for structure S-H, M06-2X/aug-cc-pVDZ level of theory.

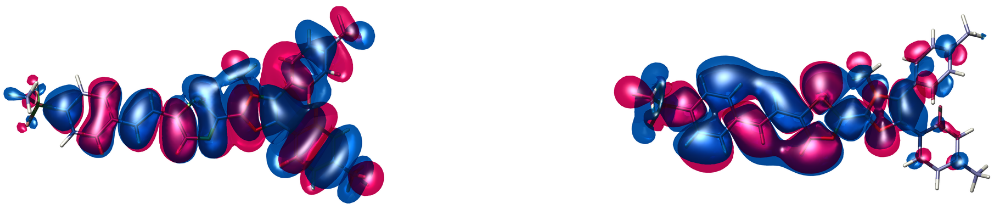

(a) HOMO (b) LUMO

Figure S6: Orbitals dominantly involved into $S_0 \rightarrow S_1$ transition for structure S-CH3, CAM-B3LYP/aug-cc-pVDZ level of theory.

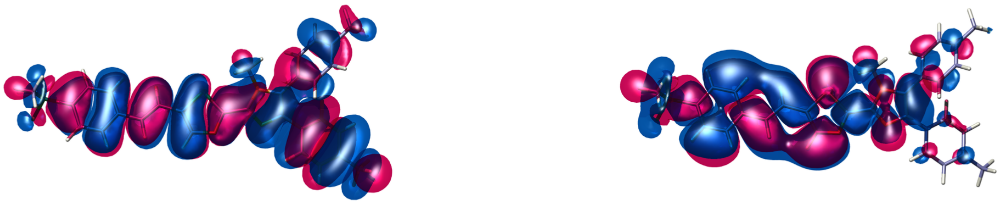

(a) HOMO−1 (b) LUMO

Figure S7: Orbitals dominantly involved into $S_0 \rightarrow S_2$ transition for structure S-CH3, CAM-B3LYP/aug-cc-pVDZ level of theory.

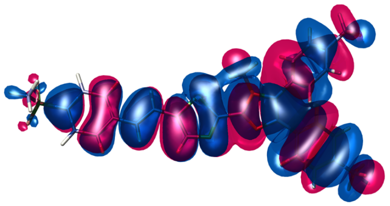

(a) HOMO

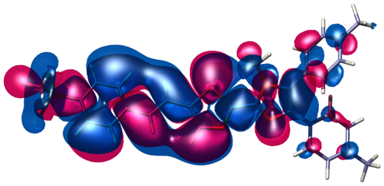

(b) LUMO

Figure S8: Orbitals dominantly involved into $S_0 \rightarrow S_1$ transition for structure S-CH3, M06-2X/aug-cc-pVDZ level of theory.

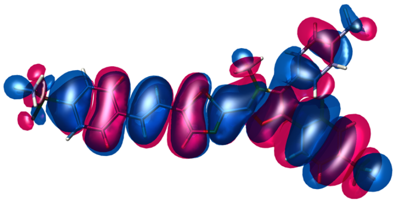

(a) HOMO$-$1

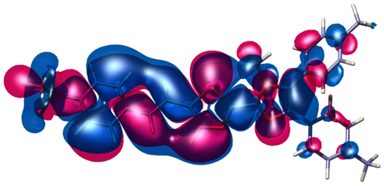

(b) LUMO

Figure S9: Orbitals dominantly involved into $S_0 \rightarrow S_2$ transition for structure S-CH3, M06-2X/aug-cc-pVDZ level of theory.

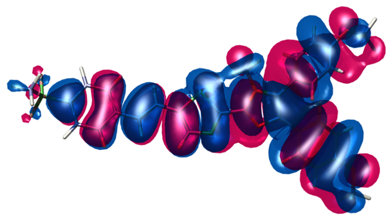

(a) HOMO

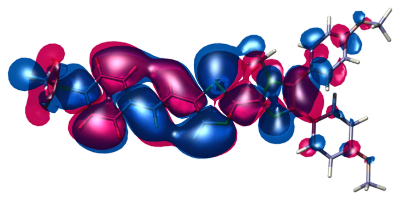

(b) LUMO

Figure S10: Orbitals dominantly involved into $S_0 \rightarrow S_1$ transition for structure S-OCH3, CAM-B3LYP/aug-cc-pVDZ level of theory.

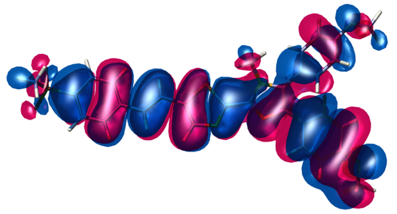

(a) HOMO-1

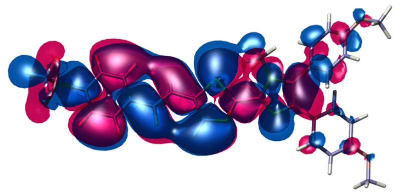

(b) LUMO

Figure S11: Orbitals dominantly involved into $S_0 \rightarrow S_2$ transition for structure S-OCH3, CAM-B3LYP/aug-cc-pVDZ level of theory.

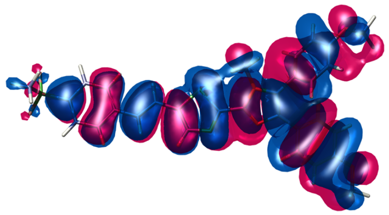

(a) HOMO

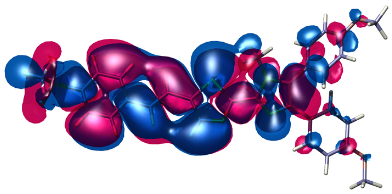

(b) LUMO

Figure S12: Orbitals dominantly involved into $S_0 \rightarrow S_1$ transition for structure S-OCH3, M06-2X/aug-cc-pVDZ level of theory.

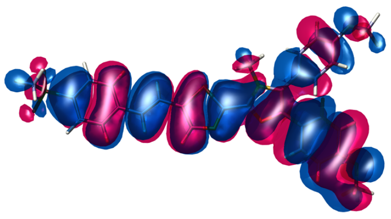

(a) HOMO−1

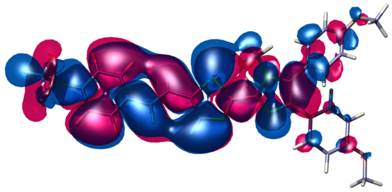

(b) LUMO

Figure S13: Orbitals dominantly involved into $S_0 \rightarrow S_2$ transition for structure S-OCH3, M06-2X/aug-cc-pVDZ level of theory.

Analysis of the spectroscopic data

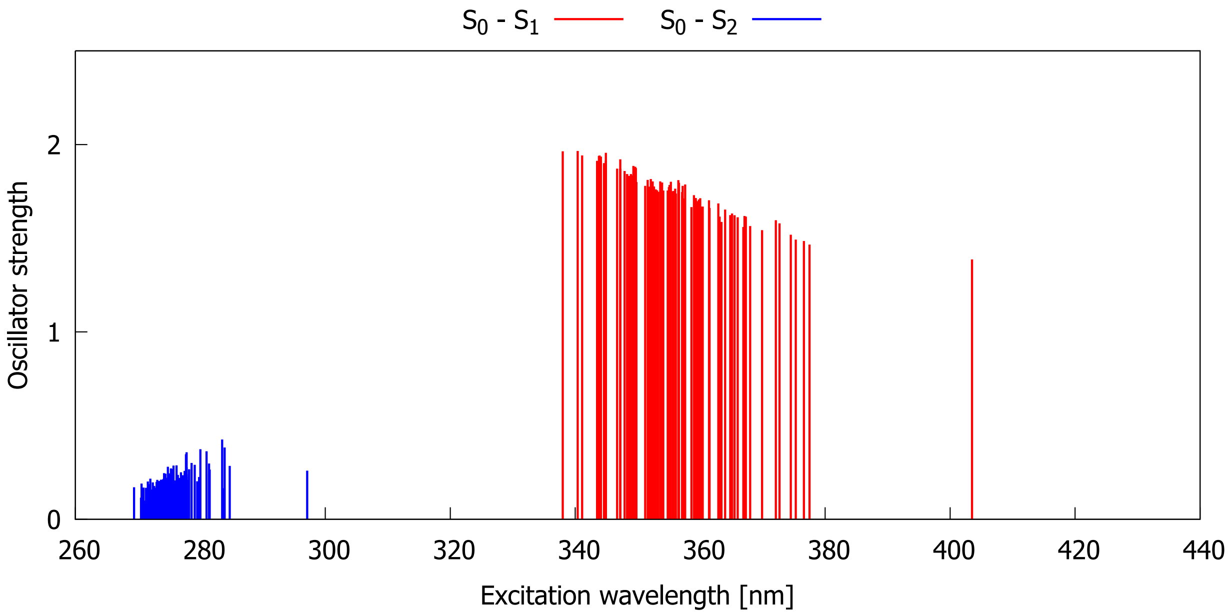

Figure S14: Simulated one-photon absorption spectrum for S-H molecule. The spectrum was obtained based on the results of calculations for 95 solute-solvent snapshots performed at the RI-CC2/cc-pVDZ level of theory.

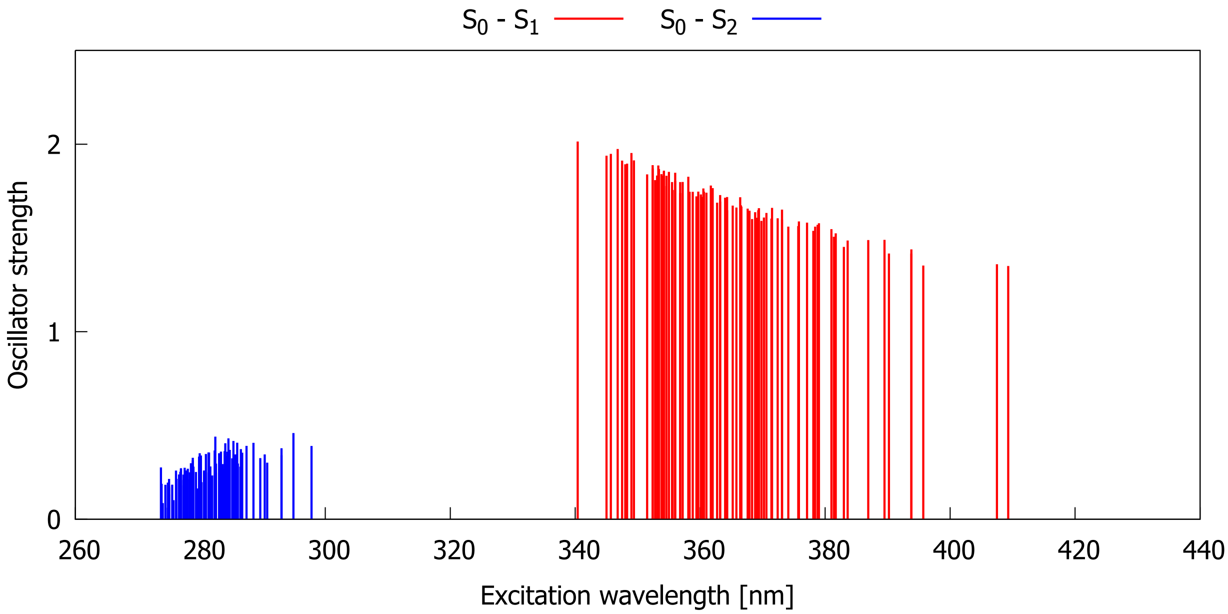

Figure S15: Simulated one-photon absorption spectrum for S-Me molecule. The spectrum was obtained based on the results of calculations for 94 solute-solvent snapshots performed at the RI-CC2/cc-pVDZ level of theory.

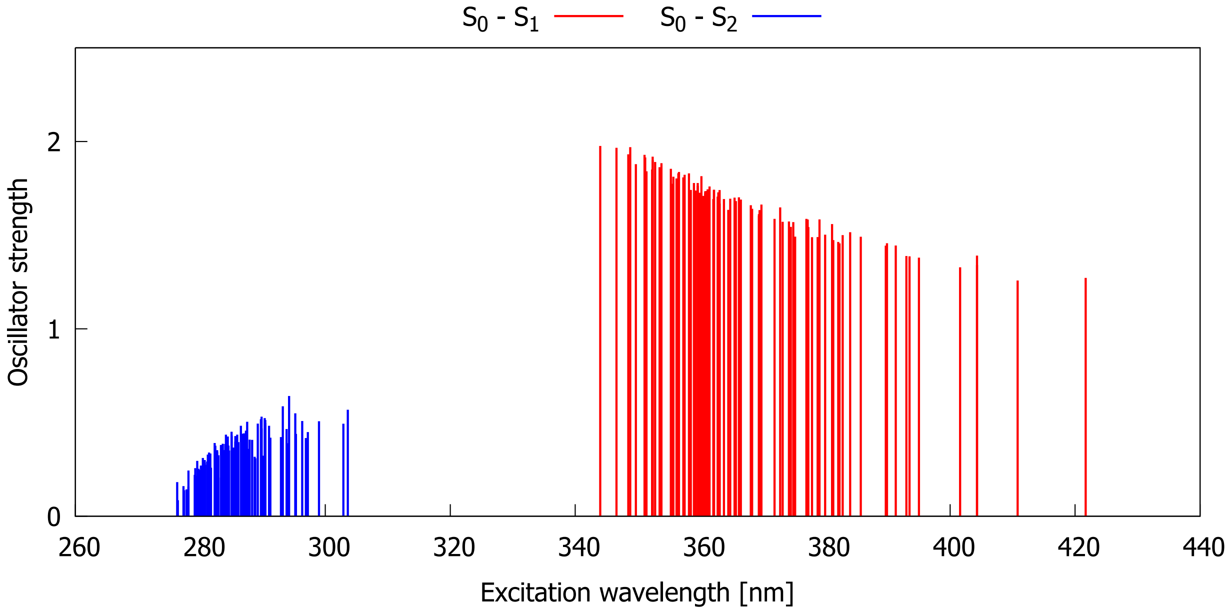

Figure S16: Simulated one-photon absorption spectrum for S-OMe molecule. The spectrum was obtained based on the results of calculations for 97 solute-solvent snapshots performed at the RI-CC2/cc-pVDZ level of theory.

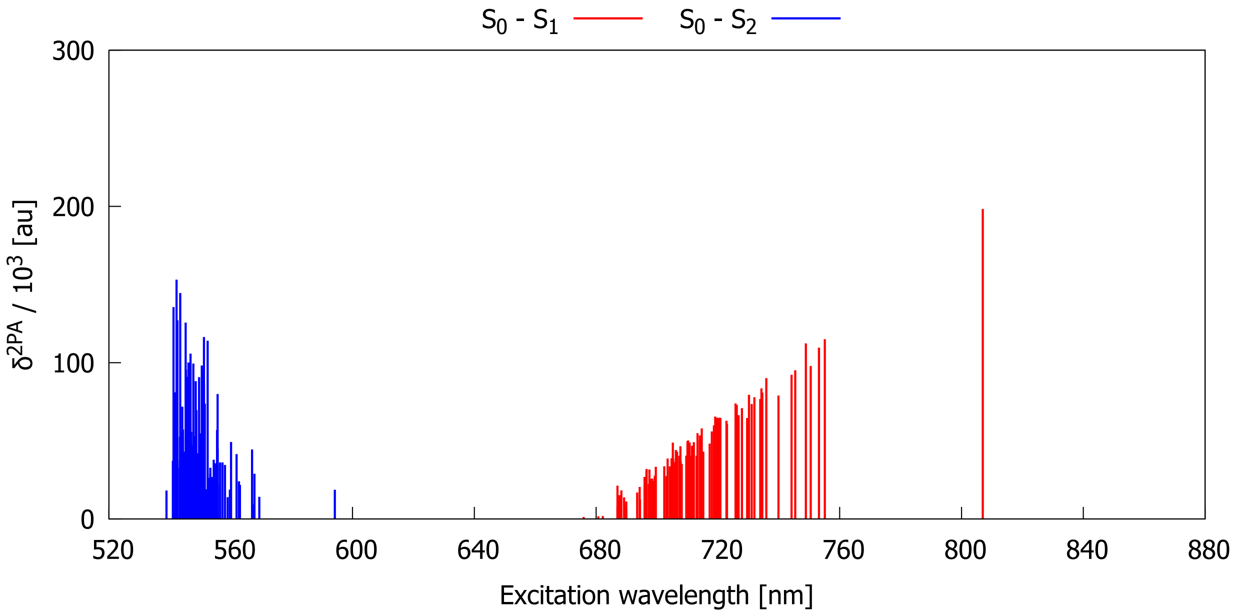

Figure S17: Simulated two-photon absorption spectrum for S-H molecule. The spectrum was obtained based on the results of calculations for 95 solute-solvent snapshots performed at the RI-CC2/cc-pVDZ level of theory.

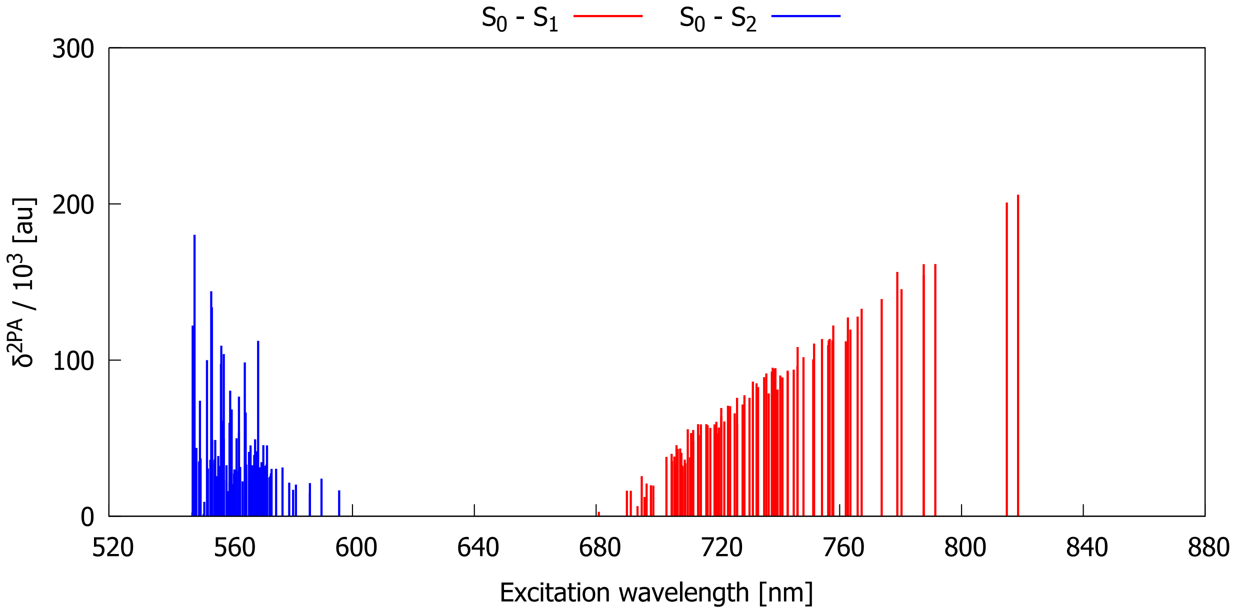

Figure S18: Simulated two-photon absorption spectrum for S-Me molecule. The spectrum was obtained based on the results of calculations for 94 solute-solvent snapshots performed at the RI-CC2/cc-pVDZ level of theory.

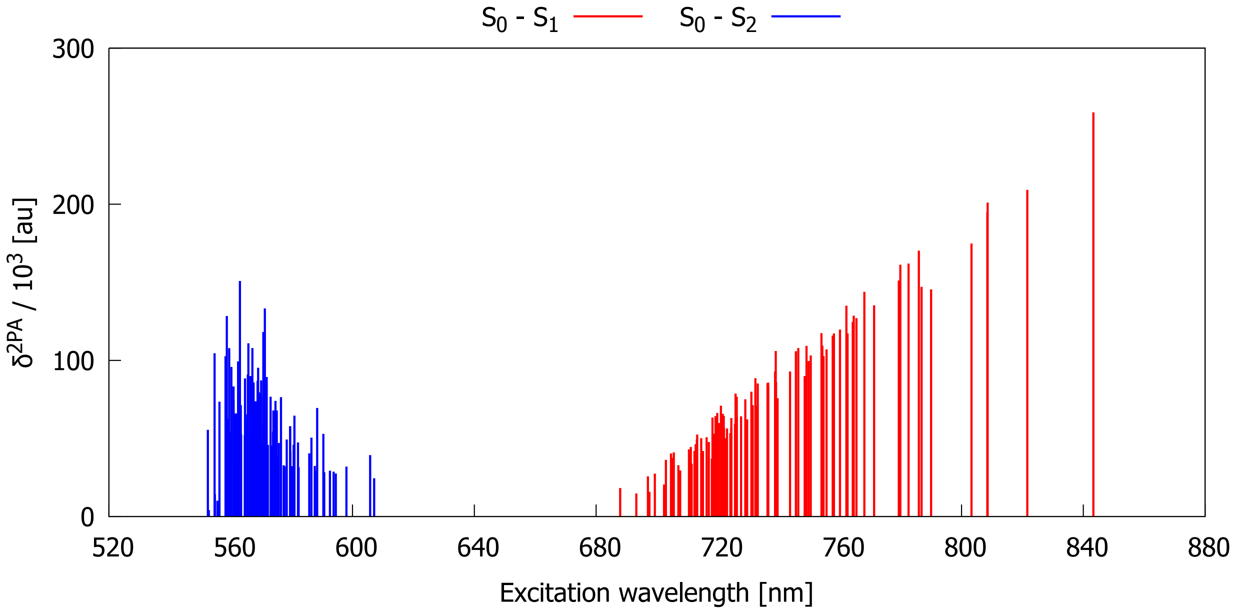

Figure S19: Simulated one-photon absorption spectrum for S-OMe molecule. The spectrum was obtained based on the results of calculations for 97 solute-solvent snapshots performed at the RI-CC2/cc-pVDZ level of theory.

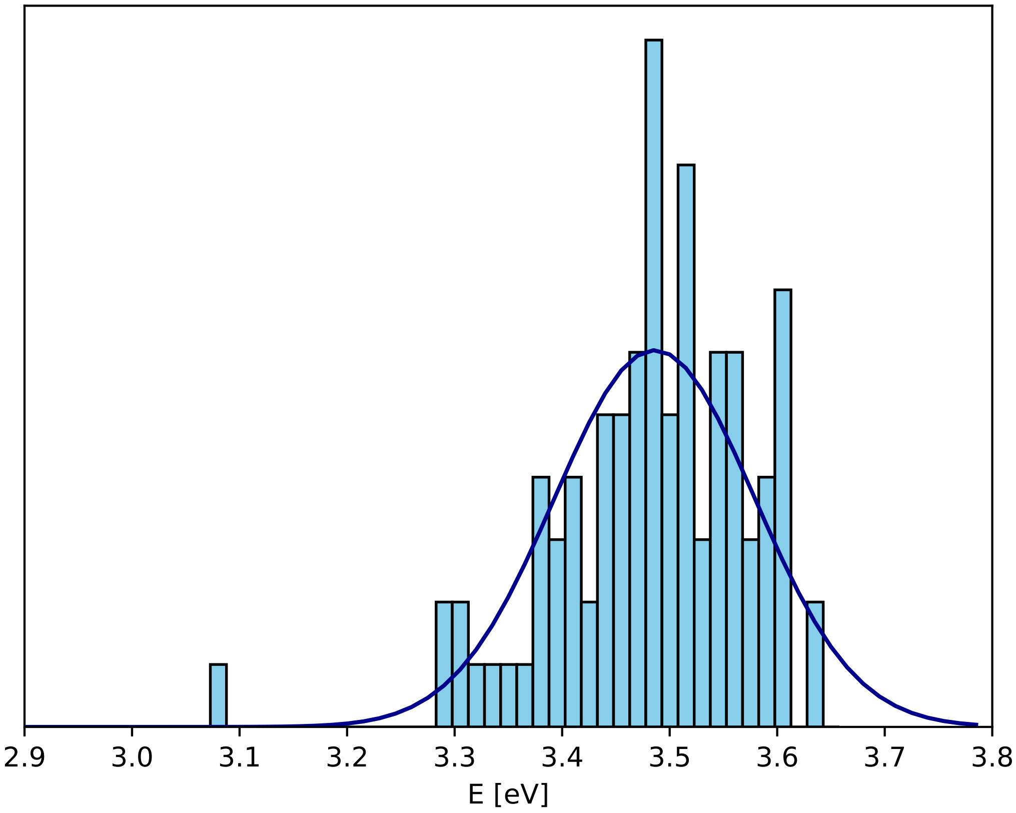

Figure S20: Distribution of vertical $S_0 \rightarrow S_1$ excitation energy for S-H molecule. Shown is the histogram of the number of solute-solvent snapshots per excitation energy interval equal to 0.015 eV.

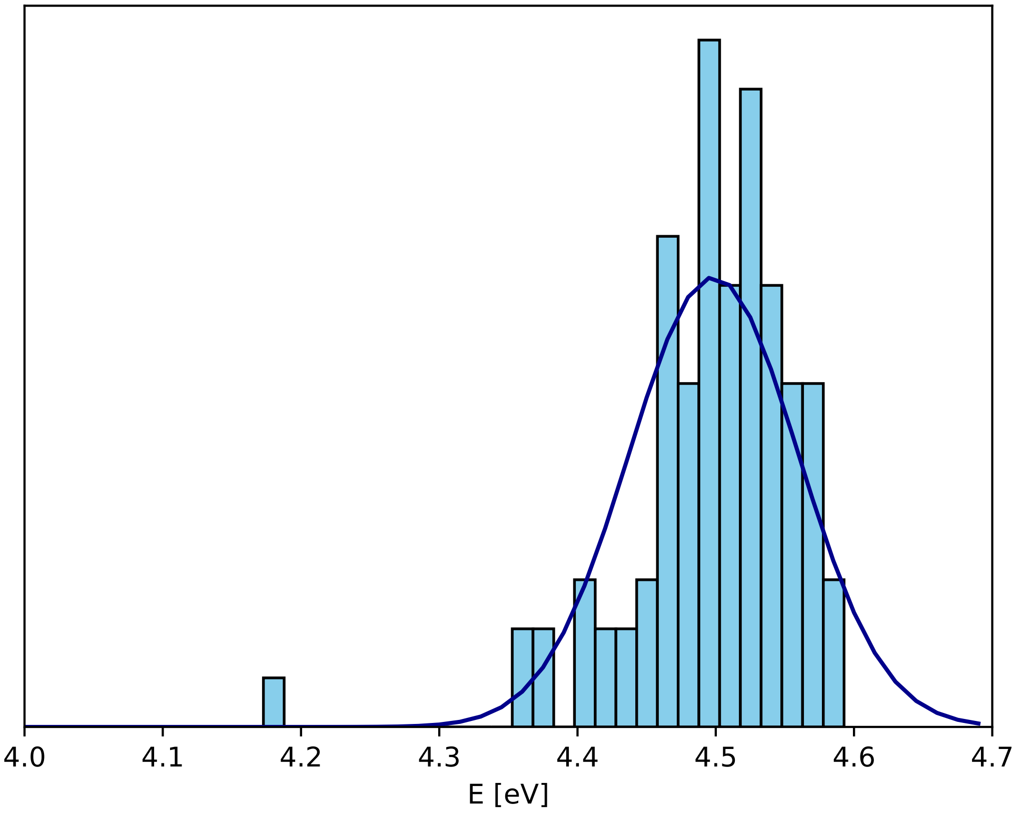

Figure S21: Distribution of vertical $S_0 \rightarrow S_2$ excitation energy for S-H molecule. Shown is the histogram of the number of solute-solvent snapshots per excitation energy interval equal to 0.015 eV.

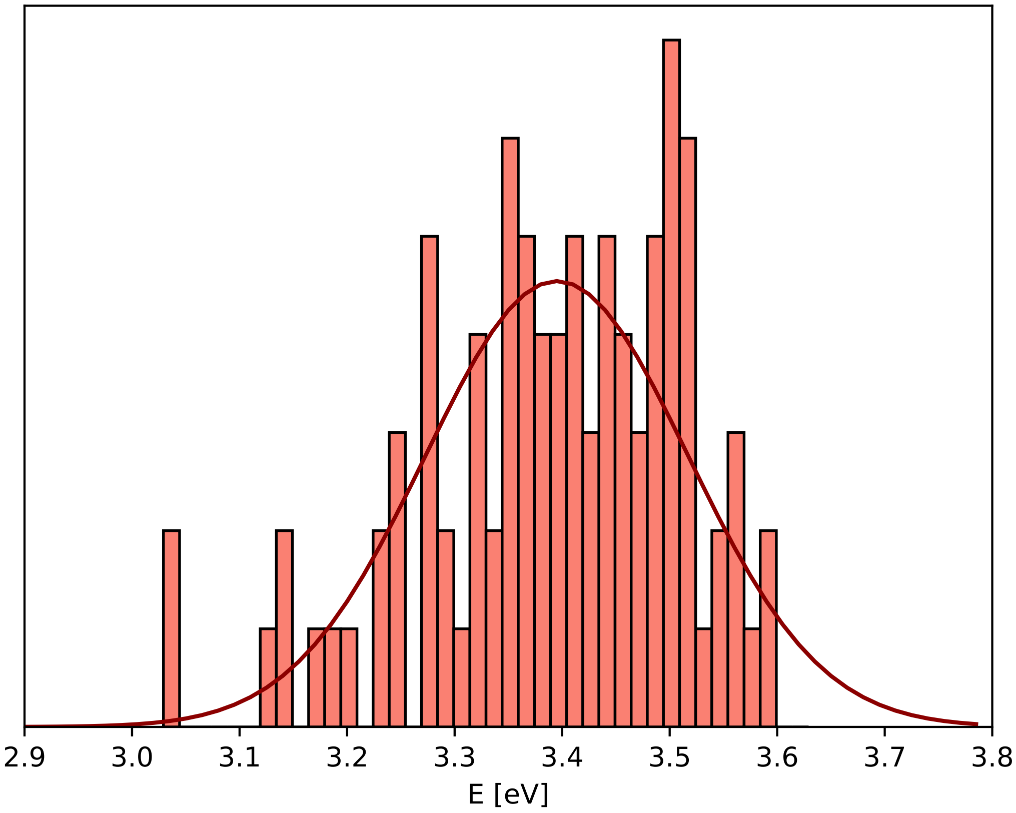

Figure S22: Distribution of vertical $S_0 \rightarrow S_1$ excitation energy for S-Me molecule. Shown is the histogram of the number of solute-solvent snapshots per excitation energy interval equal to 0.015 eV.

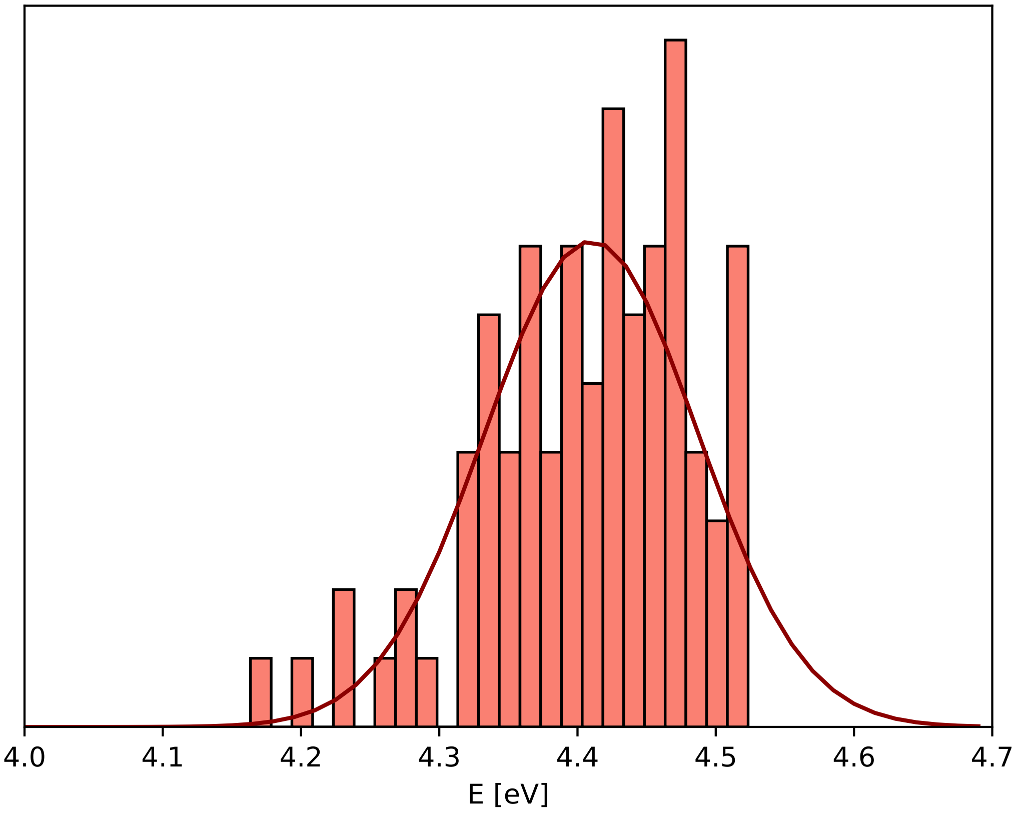

Figure S23: Distribution of vertical $S_0 \rightarrow S_2$ excitation energy for S-Me molecule. Shown is the histogram of the number of solute-solvent snapshots per excitation energy interval equal to 0.015 eV.

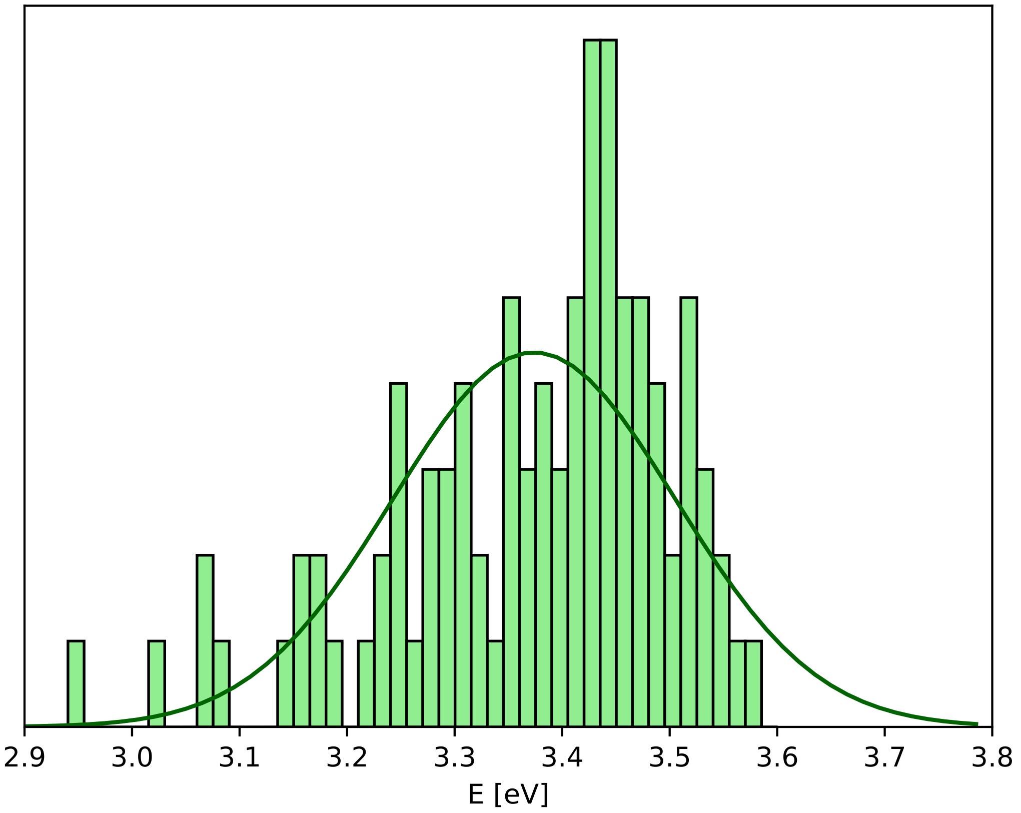

Figure S24: Distribution of vertical $S_0 \rightarrow S_1$ excitation energy for S-OMe molecule. Shown is the histogram of the number of solute-solvent snapshots per excitation energy interval equal to 0.015 eV.

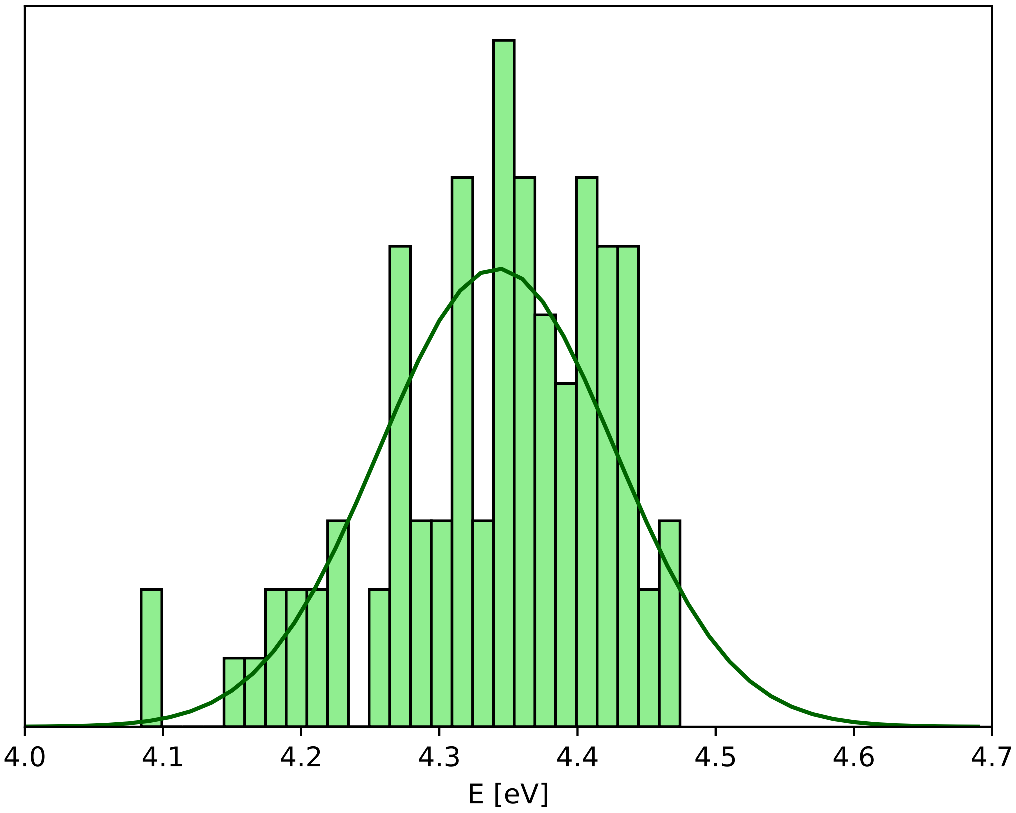

Figure S25: Distribution of vertical $S_0 \rightarrow S_2$ excitation energy for S-OMe molecule. Shown is the histogram of the number of solute-solvent snapshots per excitation energy interval equal to 0.015 eV.

Table S5: Excitation energies (ΔE, in eV), excitation wavelengths (λ, in nm), oscillator strengths (f) and two-photon transition strengths (δ^{2PA}, in au) corresponding to excitations to the two lowest-energy singlet excited states. The values were obtained at the RI-CC2/cc-pVDZ level of theory.

	$S_0 \rightarrow S_1$				$S_0 \rightarrow S_2$			
Molecule	ΔE	λ	f	δ^{2PA}	ΔE	λ	f	δ^{2PA}
S-H	3.4901	355	1.761	44496	4.5217	274	0.225	40913
S-Me	3.4030	364	1.648	77530	4.4193	281	0.260	27252
S-OMe	3.3827	367	1.690	85174	4.3494	285	0.314	38506

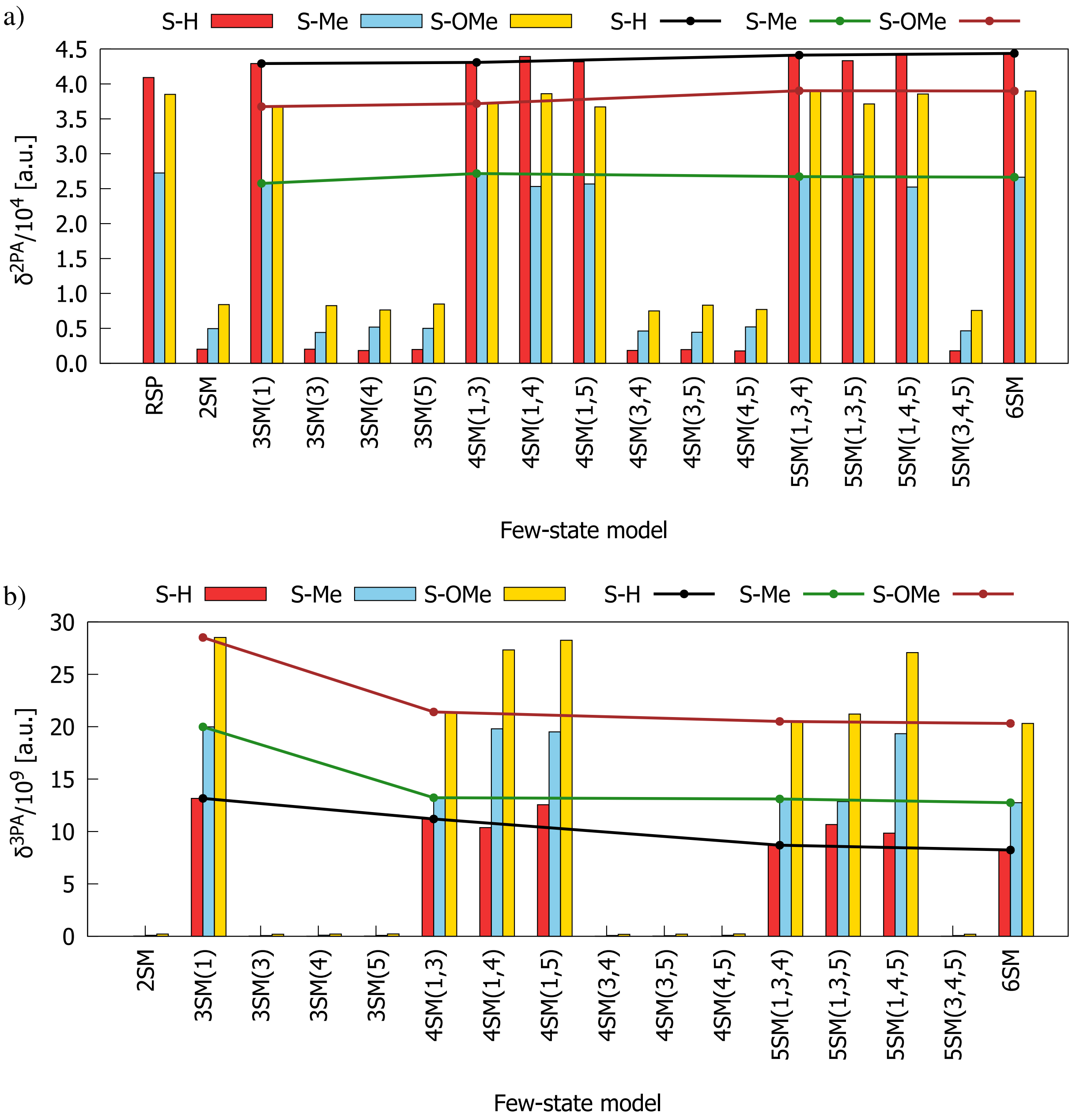

Figure S26: Comparison of response theory and few-state model results for a) 2PA and b) 3PA ($S_0 \rightarrow S_2$ transition). Solid lines show δ^{2PA} and δ^{3PA} convergence with states.

a)

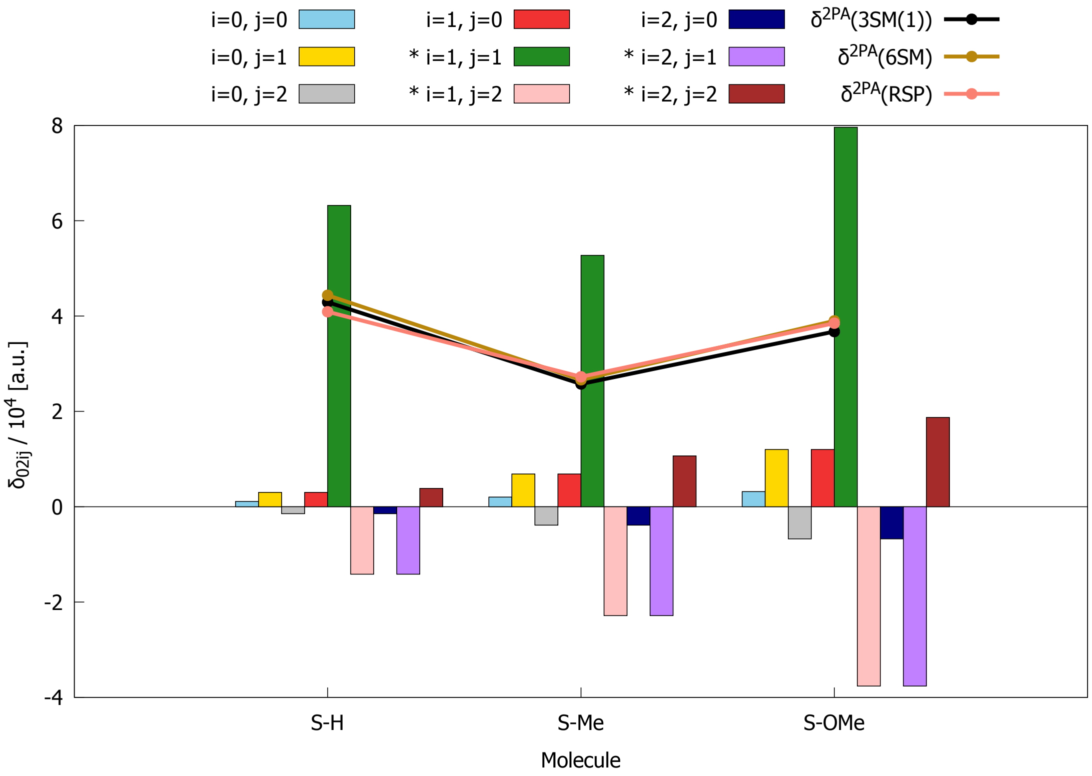

b)

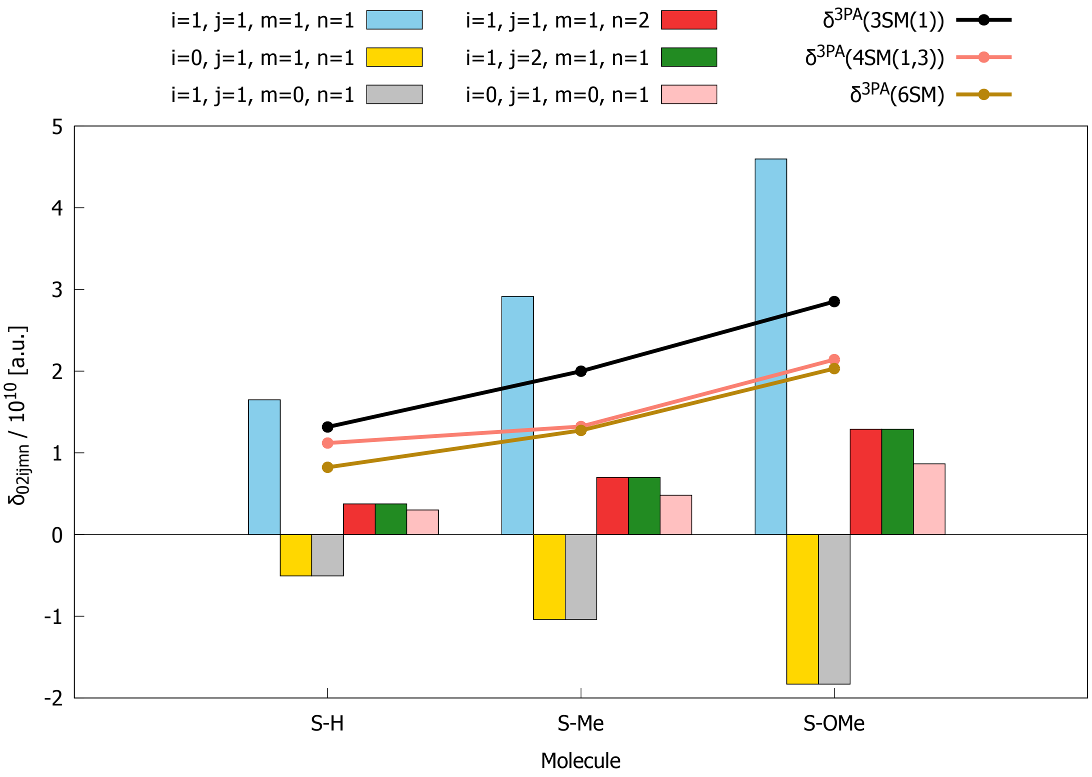

Figure S27: a) Components of $\delta^{2\mathrm{PA}}(\mathrm{S}_0 \rightarrow \mathrm{S}_2)$ within 3SM(1). * denotes the highest values of δ_{02ij} among all components of $\delta^{2\mathrm{PA}}(\mathrm{S}_0 \rightarrow \mathrm{S}_2)$ within 3SM(1), 4SM(1,3), 4SM(1,4) and 4SM(1,5) models. b) Six highest $\delta_{02\mathrm{ijmn}}$ values among all components of $\delta^{3\mathrm{PA}}(\mathrm{S}_0 \rightarrow \mathrm{S}_2)$ within 3SM(1), 4SM(1,3), 4SM(1,4) and 4SM(1,5) models.

a)

b)

c)

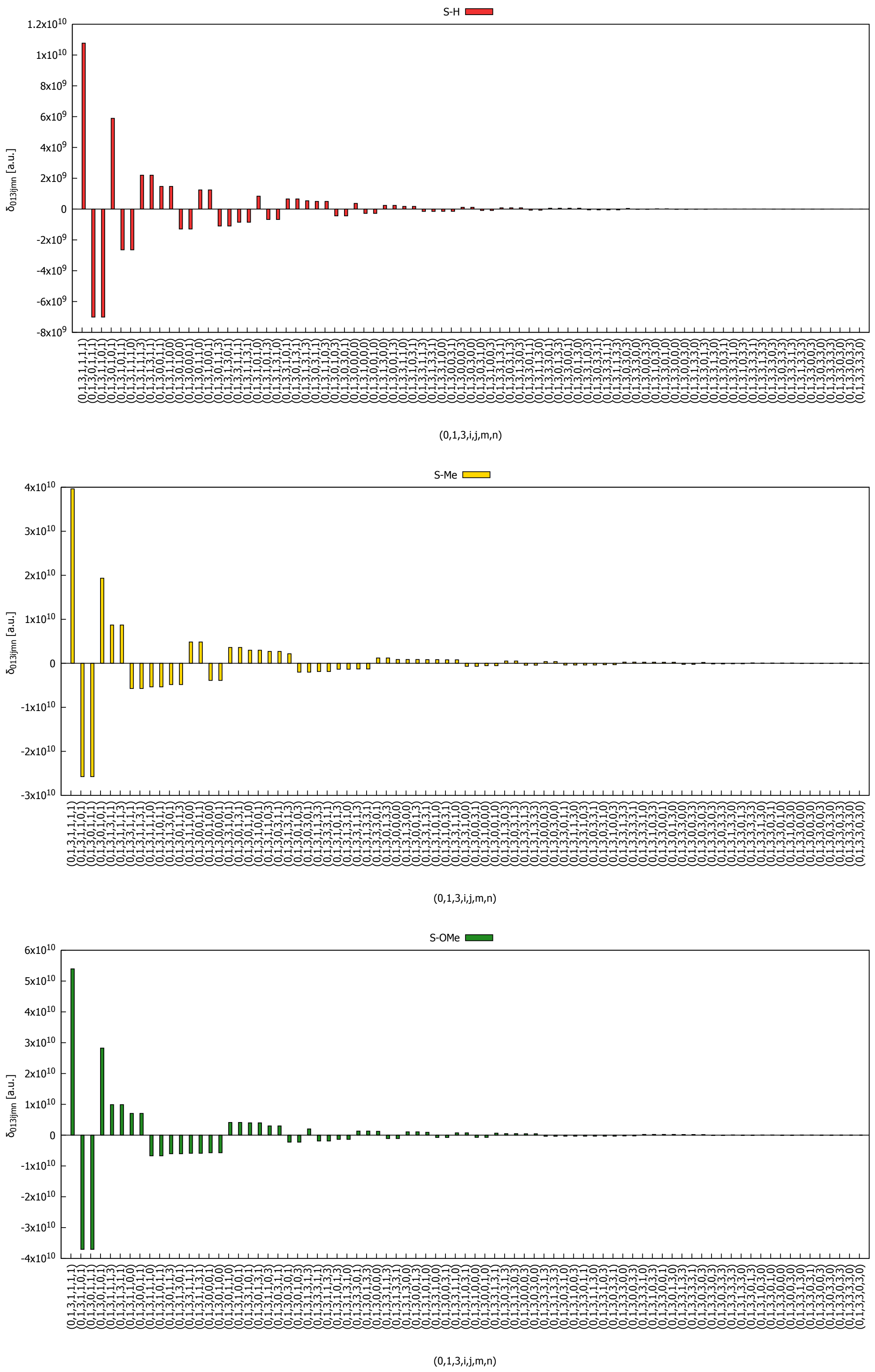

Figure S28: Components of $\delta^{3PA}(S_0 \rightarrow S_1)$ of a) S-H b) S-Me c) S-OMe molecule within 3SM(3).

a)

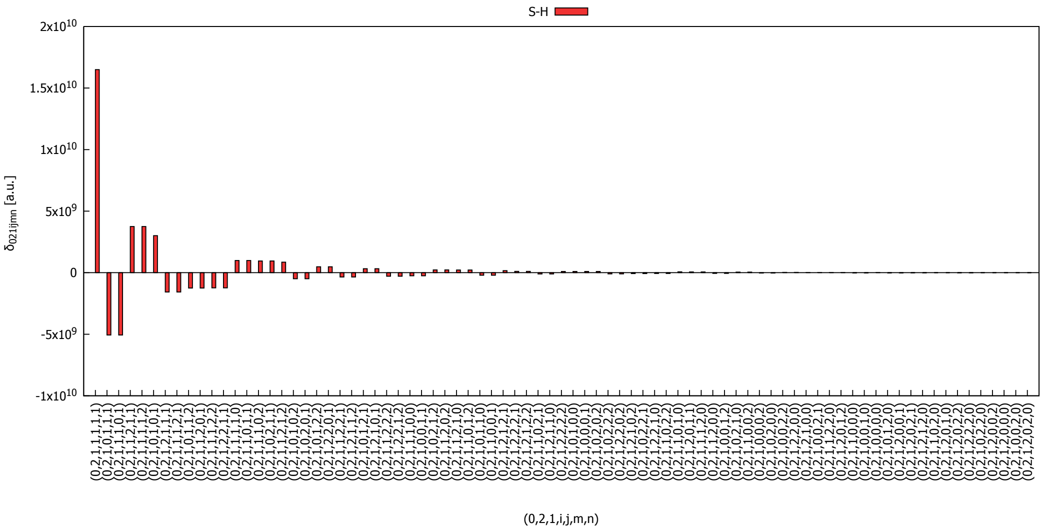

b)

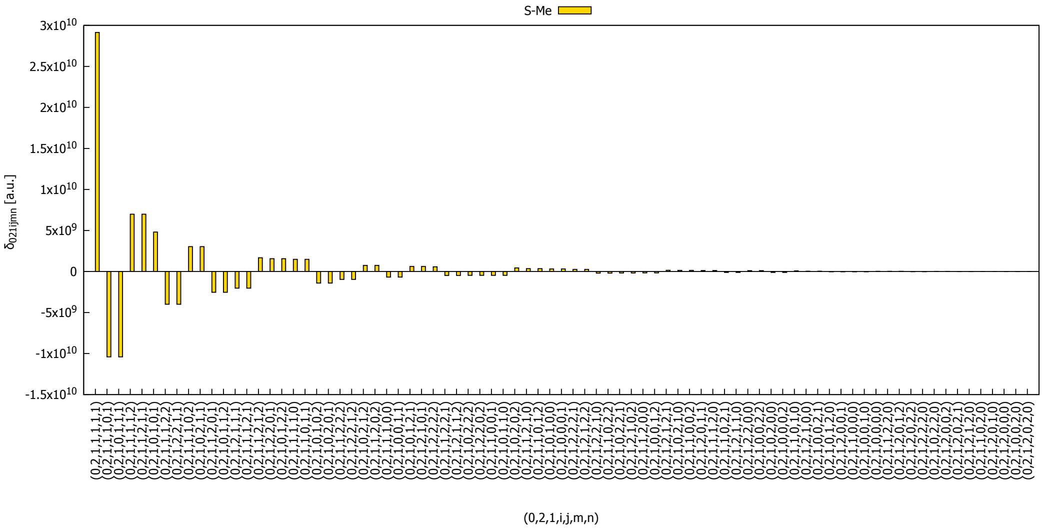

c)

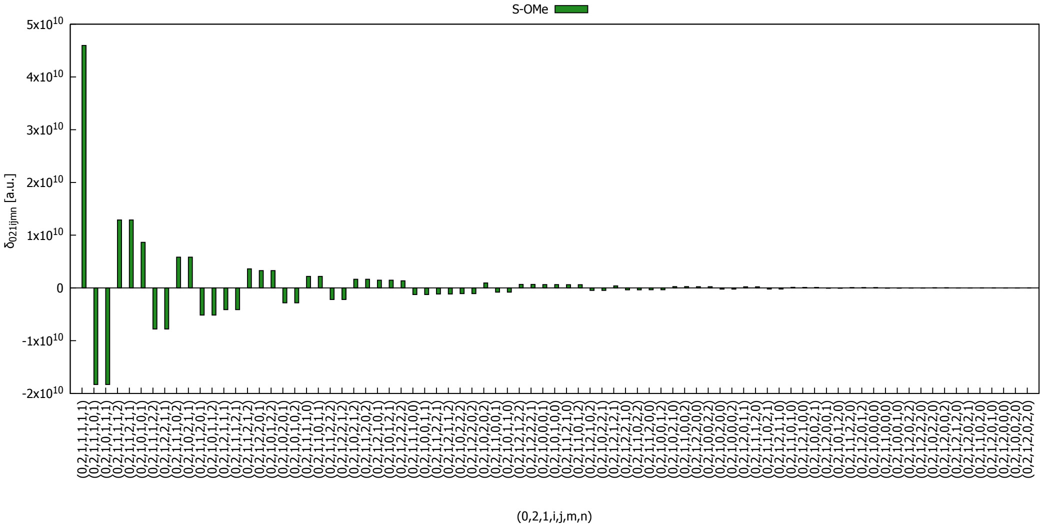

Figure S29: Components of $\delta^{3\mathrm{PA}}(\mathrm{S}_0 \rightarrow \mathrm{S}_2)$ of a) S-H b) S-Me and c) S-OMe molecule within 3SM(1).

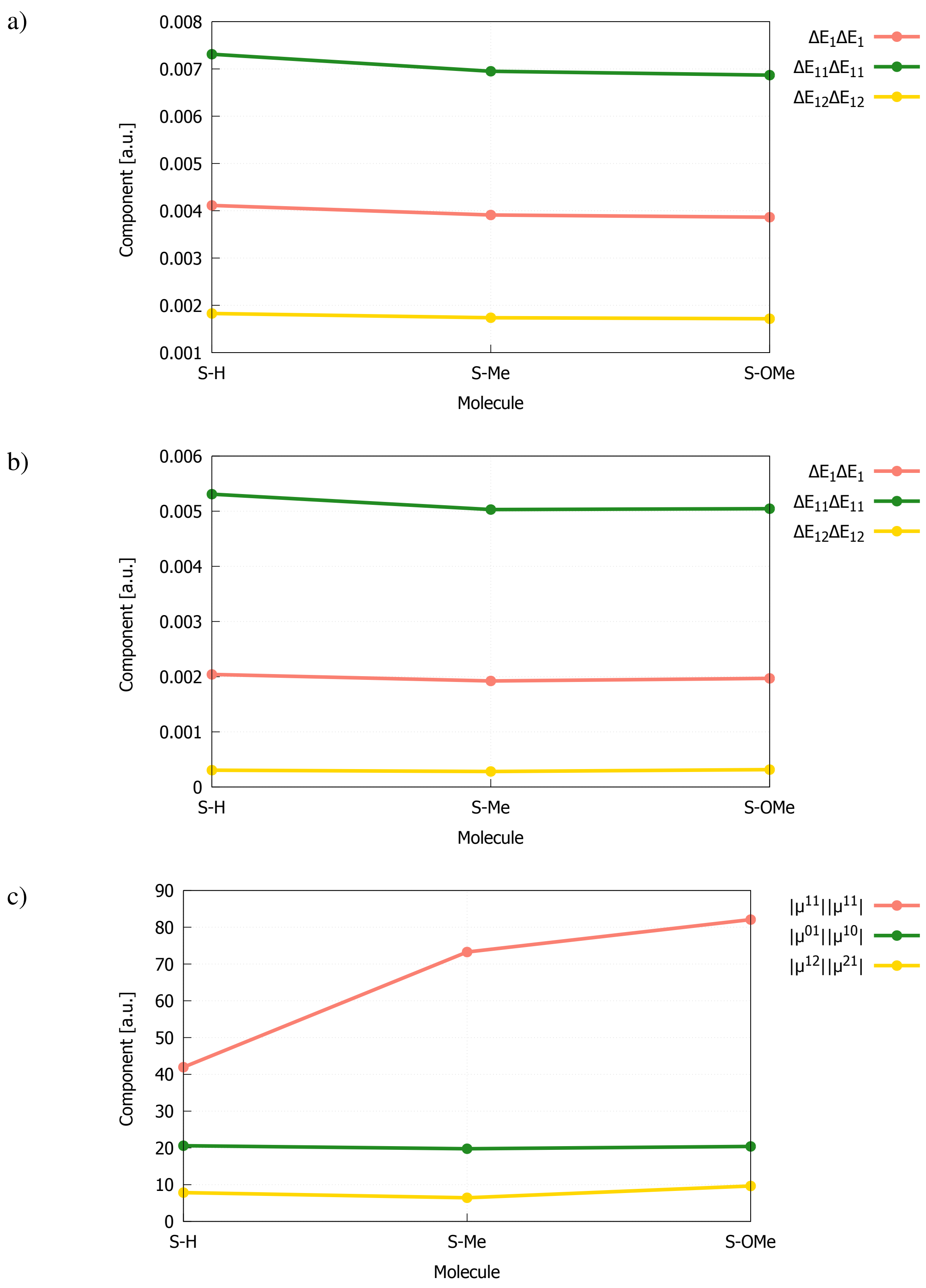

Figure S30: Energy (a for $S_0 \rightarrow S_1$ and b for $S_0 \rightarrow S_2$) and dipole (c) components of δ_{0111}, δ_{0211}, δ_{011111} and δ_{021111} ($\delta_{0111} = \frac{2|\mu^{10}||\mu^{01}||\mu^{11}||\mu^{11}|}{\Delta E_1 \Delta E_1} \cdot \mathrm{AT}$, $\delta_{0211} = \frac{2|\mu^{12}||\mu^{21}||\mu^{01}||\mu^{10}|}{\Delta E_1 \Delta E_1} \cdot \mathrm{AT}$, $\delta_{011111} = \frac{2|\mu^{11}|^4|\mu^{10}||\mu^{01}|}{\Delta E_{11}^2 \Delta E_{12}^2} \cdot \mathrm{AT}$, $\delta_{021111} = \frac{2|\mu^{11}|^2|\mu^{10}||\mu^{01}||\mu^{12}||\mu^{21}|}{\Delta E_{11}^2 \Delta E_{12}^2} \cdot \mathrm{AT}$, AT - angular part)

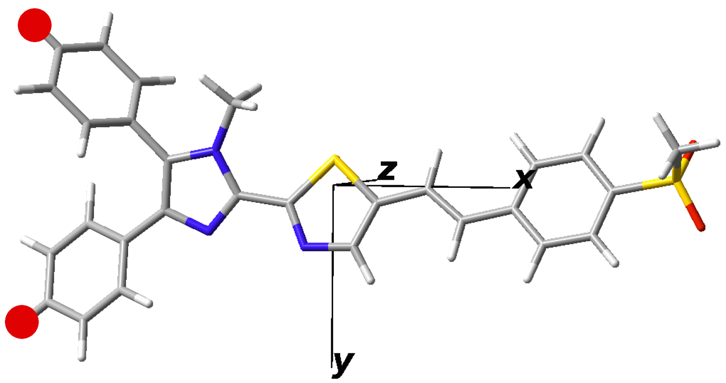

Figure S31: Orientation of studied molecules in cartesian coordinate system used for property calculations (see Table S6).

Table S6: Dipole moment components (in au) in two electronic states computed at CC2/cc-pVDZ level.

	S_0			S_1		
	x	y	z	x	y	z
S-H	-0.87	-2.94	-2.05	-5.41	-2.94	-2.01
S-Me	-1.89	-2.93	-1.99	-7.80	-2.92	-1.95
S-OMe	-2.34	-2.57	-2.26	-8.41	-2.54	-2.20

References

(1) Alam, M. M.; Beerepoot, M. T. P.; Ruud, K. Channel Interference in Multiphoton Absorption. *J. Chem. Phys.* **2017**, *146*, 244116.

(2) Hättig, C.; Christiansen, O.; Jo/rgensen, P. Multiphoton Transition Moments and Absorption Cross Sections in Coupled Cluster Response Theory Employing Variational Transition Moment Functionals. *J. Chem. Phys.* **1998**, *108*, 8331–8354.

(3) Pedretti, A.; Mazzolari, A.; Gervasoni, S.; Fumagalli, L.; Vistoli, G. The VEGA Suite of Programs: A Versatile Platform for Cheminformatics and Drug Design Projects. *Bioinformatics* **2020**,

(4) Phillips, J. C.; Braun, R.; Wang, W.; Gumbart, J.; Tajkhorshid, E.; Villa, E.; Chipot, C.; Skeel, R.; Kalé, L.; Schulten, K. Scalable Molecular Dynamics with NAMD. *J. Comput. Chem.* **2005**, *26*, 1781–1802.

(5) MacKerell, A. D.; Bashford, D.; Bellott, M.; Dunbrack, R. L.; Evanseck, J. D.; Field, M. J.; Fischer, S.; Gao, J.; Guo, H.; Ha, S.; Joseph-McCarthy, D.; Kuchnir, L.; Kuczera, K.; Lau, F. T. K.; Mattos, C.; Michnick, S.; Ngo, T.; Nguyen, D. T.; Prodhom, B.; Reiher, W. E.; Roux, B.; Schlenkrich, M.; Smith, J. C.; Stote, R.; Straub, J.; Watanabe, M.; Wiórkiewicz-Kuczera, J.; Yin, D.; Karplus, M. All-Atom Empirical Potential for Molecular Modeling and Dynamics Studies of Proteins. *J. Phys. Chem. B* **1998**, *102*, 3586–3616, PMID: 24889800.

(6) others,, et al. CHARMM General Force Field: A Force Field for Drug-Like Molecules Compatible with the CHARMM All-Atom Additive Biological Force Fields. *J. Comput. Chem.* **2010**, *31*, 671–690.

(7) Strader, M.; Feller, S. A Flexible All-Atom Model of Dimethyl Sulfoxide for Molecular Dynamics Simulations. *J. Phys. Chem. A* **2002**, *106*, 1074–1080.

(8) TURBOMOLE V7.3 2018, a development of University of Karlsruhe and Forschungszentrum Karlsruhe GmbH, 1989-2007,TURBOMOLE GmbH, since 2007; available from `http://www.turbomole.com`.
